# Genotype Specific Photosynthesis x Environment Interactions Captured by Automated Fluorescence Canopy Scans Over Two Fluctuating Growing Seasons

**DOI:** 10.3389/fpls.2019.01482

**Published:** 2019-11-20

**Authors:** Beat Keller, Shizue Matsubara, Uwe Rascher, Roland Pieruschka, Angelina Steier, Thorsten Kraska, Onno Muller

**Affiliations:** ^1^IBG-2: Plant Sciences, Institute of Bio- and Geosciences, Forschungszentrum Jülich GmbH, Jülich, Germany; ^2^Field Lab Campus Klein-Altendorf, University of Bonn, Rheinbach, Germany

**Keywords:** cold acclimation, photosynthesis x environment interactions, electron transport, chlorophyll fluorescence, high-throughput phenotyping

## Abstract

Photosynthesis reacts dynamic and in different time scales to changing conditions. Light and temperature acclimation balance photosynthetic processes in a complex interplay with the fluctuating environment. However, due to limitations in the measurements techniques, these acclimations are often described under steady-state conditions leading to inaccurate photosynthesis estimates in the field. Here we analyze the photosynthetic interaction with the fluctuating environment and canopy architecture over two seasons using a fully automated phenotyping system. We acquired over 700,000 chlorophyll fluorescence transients and spectral measurements under semi-field conditions in four crop species including 28 genotypes. As expected, the quantum efficiency of the photosystem II (F_v_/F_m_ in the dark and F_q_'/F_m_' in the light) was determined by light intensity. It was further significantly affected by spectral indices representing canopy structure effects. In contrast, a newly established parameter, monitoring the efficiency of electron transport (F_r2_/F_v_ in the dark respective F_r2_'/F_q_' in the light), was highly responsive to temperature (R^2^ up to 0.75). This parameter decreased with temperature and enabled the detection of cold tolerant species and genotypes. We demonstrated the ability to capture and model the dynamic photosynthesis response to the environment over entire growth seasons. The improved linkage of photosynthetic performance to canopy structure, temperature and cold tolerance offers great potential for plant breeding and crop growth modeling.

## Introduction

Photosynthesis is a highly dynamic process responding to environmental changes, especially to light intensity and temperature, in short- and long-term acclimation (Demmig-Adams et al., 2012; Kono and Terashima, 2014). Excess light energy is dissipated within seconds upon thylakoid lumen acidification, known as non-photochemical quenching (NPQ) (Noctor et al., 1991; Adams III et al., 2006; Croce, 2015). This protection status can last or relaxes within minutes (Demmig-Adams et al., 2012; Kromdijk et al., 2016). In contrast, acclimation and deacclimation to low temperature is slower requiring up to a few days (Huner et al., 1993; Rapacz et al., 2008; Zuther et al., 2015). Photosynthetic performance at low temperature is tightly linked to cold tolerance (Rapacz et al., 2008; Dahal et al., 2012). Traits reflecting cold tolerance in the field are an important breeding target in order to adapt crop productivity to a colder and changing climate (Rapacz et al., 2014; Frascaroli, 2018). However, photosynthetic regulation under natural fluctuating conditions, especially in response to temperature, is hardly known (Rogers et al., 2017; Murchie et al., 2018).

Cold acclimation is initiated, amongst other factors, by an imbalance between photosynthetic energy uptake and the metabolomic sink (Huner et al., 1998; Ensminger et al., 2006; Hüner et al., 2012). At low temperatures, cold responsive genes trigger different acclimation processes regulating, e.g., the expression of hormones, chloroplast proteins, and proteins associated with lipid metabolism (Svensson et al., 2006; Liu et al., 2019). Differences in cold tolerant and sensitive barley genotypes were reported in expression of cold responsive genes and photosynthetic capacity at low temperature (Rapacz et al., 2008). Photosynthesis itself acclimates to cold temperature by synthesizing more photosynthetic proteins since enzyme activity is decreased and by maintaining greater membrane fluidity (Yamori et al., 2014). Membrane fluidity at high and chilling temperatures is adjusted by the amount of trienoic fatty acids in the chloroplast and thylakoid membrane (Mitchell and Barber, 1986; Murakami et al., 2000; Sage and Kubien, 2007). This process ensures the optimal function of proteins integrated into the membrane and diffusion of electron carriers for photosynthetic electron transport (Upchurch, 2008; Liu et al., 2013). In consequence, temperature acclimation and chilling stress alter electron transport kinetics between photosystem II (PSII) and photosystem I (PSI) (Yamasaki et al., 2002; Suzuki et al., 2011).

Photosynthesis under field conditions is complex not only because of the fluctuating environment but also due to the canopy structure. Sunlight flecks which occur by canopy movement and light penetration into the canopy alter the photosynthetic status (Jia et al., 2013; Kono and Terashima, 2014; Townsend et al., 2018; Kaiser et al., 2018b). Consequently, photosynthetic light responses differ in controlled versus field conditions (Rascher and Nedbal, 2006; Meacham et al., 2017; Vialet-Chabrand et al., 2017). Leaves grown under fluctuating conditions showed lower photosynthetic capacity compared to controlled conditions but similar photosynthetic rates per leaf area (Vialet-Chabrand et al., 2017). In addition, leaves in the lower canopy acclimate to shading with decreasing photosynthesis and nitrogen content (Evans and Poorter, 2001; D’Odorico et al., 2019). Sunlight flecks interfere with this acclimation process and high net photosynthetic rates are maintained (Kaiser et al., 2018a).

In order to understand the dynamic regulation and acclimation of canopy photosynthesis in response to a fluctuating environment, high-throughput photosynthesis phenotyping systems are required with a high spatio-temporal resolution. Chlorophyll fluorescence (ChlF)-based measurements serve as a fast proxy for net photosynthesis derived from CO_2_ gas exchange measurements (Warren and Dreyer, 2006; Fiorani and Schurr, 2013; Kalaji et al., 2017). However, existing ChlF phenotyping platforms operate under static conditions and are not able to catch natural environmental interactions (Jansen et al., 2009; Mishra et al., 2014; Flood et al., 2016; Wang et al., 2018). Under field conditions, measurements are usually carried out manually and restricted to a few days and to the leaf level (Rapacz et al., 2004; Ribeiro et al., 2004; Demmig-Adams et al., 2012; Moura dos Santos et al., 2013). ChlF-based operating efficiency of PSII (F_q_'/F_m_') is widely used to assess photosynthetic performance (Baker, 2008; Kalaji et al., 2016). F_q_'/F_m_' is sensitive to light, as it is affected by the amount of NPQ at the PSII antennae (Von Caemmerer, 2000; Croce, 2015; Lazár, 2015). However, changes in electron transport between PSII and PSI, which occur in response to temperature, are difficult to detect using F_q_'/F_m_' because it represents the efficiency of charge separation in the PSII reaction center (Van Heerden et al., 2003; Bezouw et al., 2019).

The light-induced fluorescence transient (LIFT) method monitors ChlF induction and relaxation within milliseconds from a distance using sub-saturating excitation flashlets (Kolber et al., 1998; Pieruschka et al., 2010; Osmond et al., 2017; Keller et al., 2019). The efficiency of PSII charge separation (F_q_'/F_m_') is derived from ChlF induction using fast repetition rate flashlets (Kolber et al., 1998). Electron transport rates derived by the LIFT method from several meter distance were highly correlated to pulse amplitude modulated and gas exchange measurements from close distance (Pieruschka et al., 2010). Further, the efficiency of electron transport beyond the primary quinone electron acceptor of PSII (Q_A_) in the dark (F_r_/F_v_) and light (F_r_'/F_q_') can be assessed *via* ChlF relaxation using flashlets with decreasing repletion rate. F_r_/F_v_ serves as a fast and robust approximation of electron transport kinetics (Keller et al., 2019). From a series of saturating flashes in the dark and the followed ChlF relaxation, it was observed that Q_A_^−^ oxidation time constants are approximately 0.2 ms to reduce the secondary quinone electron acceptor (Q_B_) and 0.7 ms to reduce Q_B_^−^ to Q_B_^2−^ (Vass et al., 1999; de Wijn and van Gorkom, 2001). The time constant of biding a plastoquinone (PQ) to a vacant Q_B_-binding site is about 2 to 3 ms which matches roughly the estimated turn-over time for a oxidized PQ to leave the Q_B_ pocket side as reduced plastoquinol (PQH_2_) (de Wijn and van Gorkom, 2001; Petrouleas and Crofts, 2005). In the light, the ChlF relaxation showed less pronounced phases and F_r_'/F_q_' derived thereof was rather insensitive to increasing light intensities (Keller et al., 2019). Besides, the LIFT device acquires the leaf reflectance spectrum. Spectral indices such as the photochemical reflectance index (PRI) are correlated to photosynthetic light use efficiency, chlorophyll content, and related to canopy structure (Barton and North, 2001; Shrestha et al., 2012; Wu et al., 2015; Schickling et al., 2016; Sukhova and Sukhov, 2018).

We established a fully automated LIFT high-throughput phenotyping system and monitored four crop species (barley, maize, soybean, wheat) over two growing seasons. The main hypothesis was that F_r_'/F_q_' is independent of F_q_'/F_m_' baring additional information to photosynthetic activity and its regulation under controlled and fluctuating conditions. Specifically the following hypotheses were addressed: 1) light intensity controls the PSII efficiency of charge separation (F_q_'/F_m_'). The light penetration into the canopy can be approximated by reflectance indices. In contrast, 2) F_r_/F_v_ and F_r_'/F_q_' show a strong dependency on temperature which is extenuated in winter hard species and cold tolerant genotypes. F_r_/F_v_ and F_r_'/F_q_' quantifies electron transport capacity indicating genotypic specific cold acclimation. 3) Fluctuating photosynthetic response and genotype x environment interactions can be modeled to predict photosynthetic performance for entire and future growing seasons.

For the first time, we quantified F_r_/F_v_ under controlled and fluctuating conditions. In addition, full reflectance spectra of the measured leaves were acquired to gain information about canopy structure. We show the photosynthetic response over the full growing season beyond snapshot phenotyping toward the full incorporation of genotype x environmental interactions using high-throughput data and modeling. F_r_/F_v_ proved to be a promising trait to study photosynthetic regulation and cold tolerance.

## Material and Methods

ChlF measurements were performed under controlled and semi-field conditions in five species and 29 genotypes by using the LIFT method in high-throughput.

### Controlled Conditions

*Arabidopsis* (*Arabidopsis thaliana*) Col-0 genotypes were grown at 23°C in 12/12 h day/night cycle in the growth chamber at around 150 µmol photons m^−2^ s^−1^. At 59 days after sowing (DAS), plants were subjected for four days to fluctuating temperature between 15 and 35°C. The temperature increased in the light and decreased in the dark. Temperature steps were 5°C in 2 h intervals followed by 4 h at 20°C. The air humidity in the climate chamber was kept at around 50–70%.

### Semi-Field Growth Conditions

The *Miniplot* facility with an automated measuring platform is located at the Field Campus Klein Altendorf (University of Bonn, Germany, 50°37′ N, 6°59′ E) in an unheated glasshouse without additional lighting (Thomas et al., 2018). The *Miniplot* facility hosts a total of 90 growth containers (111 x 71 x 61 cm) with a volume of 535 L filled with a loamy-clay silt soil (luvisol) from the nearby field site (Hecht et al., 2016). Containers were drip irrigated with approximately 16 L per week. The amount was increased to up to 36 L per week in hot weather conditions.

#### Soybean

Soybean [*Glycine max* (L.) Merr.] genotypes differing in cold tolerance were kindly provided by the Swiss soybean breeding program of Agroscope (Changins, Switzerland). Genotypes Amarok, Gallec, and Tourmaline are tolerant to cold whereas 22216, S1, and Protibus are cold sensitive (**Supplementary Table 1**) (Gass et al., 1996). In 2016, soybean genotypes were sown on August 19 directly into the containers of the *Miniplot* facility. 22 seeds per container were sown 3 cm deep in two rows (distance 40 cm) every 10 cm. Five genotypes in two replicates and one genotype (S1) in 1 replicate were planted in 11 randomized containers. On September 20, each container was fertilized with 30 g Hakaphos^®^ Blue (N-P-K, 1.0–0.7–1.0, COMPO EXPERT GmbH, Münster, Germany).

In 2017, Genotype Protibus was excluded Bahia, Eiko, and MinnGold were included into the trial. The MinnGold genotype has a chlorophyll-deficient phenotype caused by a spontaneous mutation in the Mg-chelatase subunit gene (ChlI1a) (Campbell et al., 2015). These eight soybean genotypes were cultivated in a greenhouse for two weeks at approximately 20°C. Then on March 23, plants were transplanted into containers in the *Miniplot*. Sixteen plants per container were arrayed into two rows (40 cm row distance). Six genotypes in four replicates and two genotypes (Bahia and Eiko) in two replicates were planted in 28 containers in a randomized block design. At 34 DAS, plants were fertilized using 24 g Hakaphos^®^ blue (COMPO EXPERT GmbH) per plot (around 3.6 g N per plot or 0.2 g N per plant). The LIFT instrument beam was focused at 1.4 m until June 21, 2017 and then adjusted to 1.2 m.

#### Maize

Five maize (*Zea mays*) genotypes of the German Plant Phenotyping Network (DPPN) reference collection were sown on May 24, 2016 (**Supplementary Table 2**). Genotypes were grown in 10 containers in a randomized block design (two containers per genotype). In 2017, nine genotypes of the DPPN reference were sown on May 30 into 18 containers.

#### Barley

Six commercial available barley (*Hordeum vulgare* L.) cultivars (Gesine, Eileen, Irina, Tocada, Grace, and Milford) were selected (Bundessortenamt, 2013). These cultivars were sown on September 16, 2016 in one container per genotype and grown as described in Thomas et al. (2018). The sowing density per plot was 360 seeds.

#### Wheat

Three wheat (*Triticum aestivum* L.) genotypes (Brilhante, PF37 and PF62) were used in this study (Poersch-Bortolon et al., 2016). Fifty seeds per meter in 15 cm row distance (five rows per container) were sown on May 12, 2016 into six containers (two containers per genotype) in a randomized block design. On June 15, 30 g Hakaphos^®^ Blue (COMPO EXPERT GmbH) per container was applied.

### Environmental Data

Environmental data were recorded every minute from three sensor stations distributed in the *Miniplot* facility. Data were uploaded to an SQL database. The used sensors were LI-190 (LI-COR Inc., Nebraska USA) for photosynthetic photon flux density (PPFD) and HMP110 (Vaisala, Helsinki, Finland) for air temperature and humidity. Environmental data were linked to LIFT measurements taken in the same minute.

### Light-Induced Fluorescence Transient Device

**Figure 1:**
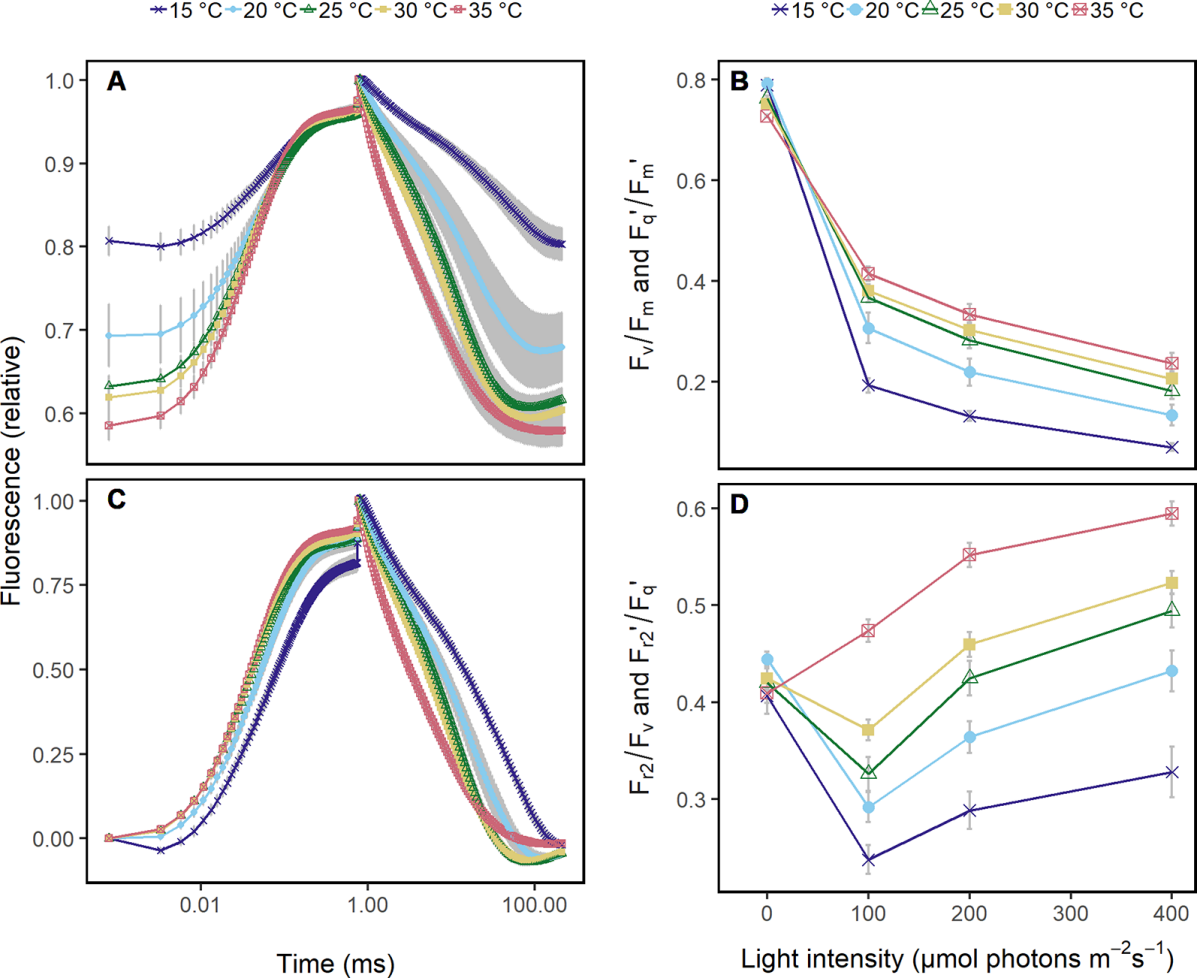
Chlorophyll fluorescence transients were acquired in *Arabidopsis* leaves from 60 cm distance using fast repetition rate excitation flashlets. Leaves were subjected to different temperatures (15 to 35°C) and different light intensities under controlled laboratory conditions. **(A)** Transient was normalized to the maximum chlorophyll fluorescence which allows to compare the quantum efficiency of the photosystem II (F_v_/F_m_ in the dark and F_q_'/F_m_' in the light) at the different temperatures. The ambient light intensity was 200 μmol photons m^−2^ s^−1^. **(B)** F_v_/F_m_ and F_q_'/F_m_' in response to increasing light intensities at the different temperatures are shown. **(C)** Chlorophyll fluorescence transients in (A) were double normalized to the maximum and minimum chlorophyll fluorescence, which allows to retrieve the efficiency of electron transport 5 ms after reduction of the primary quinone (Q_A_) (F_r2_/F_v_ in the dark, F_r2_'/F_q_' in the light). **(D)** F_r2_/F_v_ and F_r2_'/F_q_' are shown in response to increasing light intensities at the different temperatures. Attached *Arabidopsis* leaves (n = 6) were measured in dark-adapted and steady-state at 0, 100, 200, and 400 μmol photons m^−2^ s^−1^ of blue light (445 nm). Error bars indicate the 95% confident interval.

The compact LIFT instrument (Version LIFT-REM, Soliense Inc., New York, USA) is equipped with a blue light-emitting diode (LED) (445 nm), a STS-VIS spectrometer (Ocean Optics, Florida, USA), and two RGB cameras (FLIR Integrated Imaging Solutions Inc., British Colombia, Canada). Subsaturating actinic LED flashlets in fast repetition rate (FRR) induce the maximum fluorescence yield and monitor its relaxation with decreasing repetition rates. ChlF is detected at 685 ( ± 10) nm. The FRR flash was used with a excitation phase of 0.75 ms (FRRF_0.75ms_) consisting of 300 flashlets (Keller et al., 2019). The relaxation phase included 127 flashlets triggered at decreasing repetition rate and lasted for 200 ms (**Figure 1A**). When measuring under ambient light, background irradiation in the wavelength range of the detector is determined between the flashlets and subtracted from the ChlF yield of every flashlet. For all measurements, the excitation power at 60 cm distance was about 40,000 photons m^−2^ s^−1^ for the ChlF induction phase, as described in Keller et al. (2019).

#### Light Response Curves Under Controlled Conditions

Blue light curves at different temperatures were carried out on *Arabidopsis* plants. Plants were dark adapted for 30 min prior to measurements (n = 5 plants). The light response curve consisted of 161 FRRFs_0.75ms_. It was one FRRF_0.75ms_ in the dark-adapted state and 40 FRRFs_0.75ms_ at each light intensity level in a 1.5 s interval. Light intensities were 80, 100, 200, 400 µmol photons m^−2^ s^−1^. Plants were measured from low to high light intensities at 25°C, 35°C (63 DAS), and the following day at 20, 15, and 30°C (64 DAS). Transition between temperatures took about 20 min. LI-COR sensors were matched at every temperature step and after every second measurement.

The blue LED of the LIFT instrument was used as actinic light source (445 nm). The size of the illumination spot was around 3 cm^2^. The intensity of the blue LED was calibrated by using a quantum sensor (LI-190R, LI-COR, Inc.) at 60 cm distance. A fully expanded leaf was placed into a LI-6400XT transparent 2x3 cm chamber head (LI-COR, Inc., Nebraska USA) and measured with the LIFT instrument through the transparent film of the chamber. The air flow rate during the measurements was 300 µmol air s^−1^ and block temperature was kept at 20°C. CO_2_ concentration in the air was controlled at 400 ppm and air flow was set to 400 µmol s^−1^.

#### Automated Measurements Under Fluctuating Semi-Field Conditions

**Figure 2:**
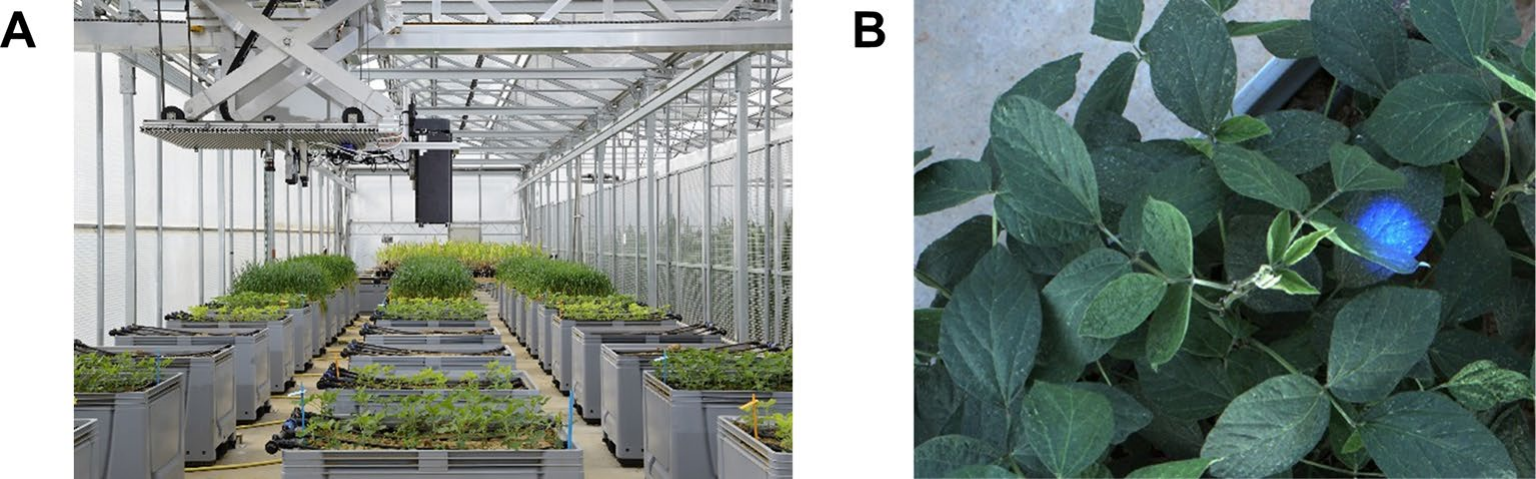
The fully automated light-induced fluorescence transient (LIFT) system scanned crop canopy in 30 mm s^−1^ measuring every 2 s. **(A)** Plants were grown in containers under fluctuating conditions in an unheated glasshouse. The measuring distance was about 1 m. **(B)** The diameter of the measuring beam on the canopy was around 30 mm.

Fully automated LIFT measurements took place from May 2016 to August 2017 using the measuring platform of the *Miniplot* facility (**Figure 2A**). Every hour, crop canopy of every container was scanned in consecutive 3 x 300 mm line measurements at a velocity of around 30 mm s^−1^ by one or two LIFT devices. The distance from the LIFT lens to soil was 1.5 m and the measurements were initially focused at 1.4 m. The focus was adjusted as plants grew. The measuring spot was around 30 mm in diameter, hence about 700 mm^2^ (**Figure 2B**). Each ChlF transient measurement took 210 ms. Every ChlF measurement was followed by a spectral measurement with 1,790 ms integration time (**Supplementary Figure 1A**). In that mode, one combined measurement was acquired every 2 s, resulting in 5 to 7 combined measurements for each of the 3 x 300 mm scans (**Supplementary Figure 2**). In total, about 18 independent measurements were acquired for each row operating with one LIFT device. The third measurement of each line was excluded since it most likely measured the same spot due to the stop of the positioning system after 300 mm. For the experiments in 2017, it was about 36 measurements since two LIFT devices operated simultaneously hanging next to each other from the moving platform.

#### Leaf Angle and Canopy Effects

In order to assess the canopy effect and different measuring distances, leaves of soybean genotype Tourmaline were fixed 30 cm aboveground with three needles on top of a bamboo stick perpendicular to the LIFT lens. Four leaves per plot in four plots were fixed (n = 16). Automated platform measured changing the distance randomized from 120 to 75 cm in 15 cm steps. Every leaf was measured with randomized distance in 14-min intervals allowing oxygen evolving complex to relax when measuring during the night.

### Fluorescence Data Processing

The ChlF transient data acquired by the LIFT FRRF_0.75ms_ represent complex processes of Q_A_ reduction and reoxidation. In order to quantitatively characterize the main processes, the ChlF transients were analyzed on an empirical basis described in Keller et al. (2019).

#### Retrieval of Minimum and Maximum Fluorescence

For the FRRF_0.75ms_ used in this study, the minimum ChlF yield F_o_ is defined as the ChlF yield of the first flashlet and the maximum ChlF yield (F_m_) as the averaged ChlF yield of the 301^st^ and 302^nd^ flashlet (Keller et al., 2019). The 300^th^ flashlet does not represent F_m_ due to quenching processes in the induction phase. The variable ChlF yield (F_v_) is the difference between F_m_ and F_o_ which was used to calculate the maximum efficiency of PSII (F_v_/F_m_) in the dark-adapted state. The light adapted states of F_v_ and F_m_ were denoted as F_q_' and F_m_.'

#### Q_A_^−^ Reoxidation Efficiency

The ChlF parameter F_r_/F_v_ represents the efficiency of reoxidation after Q_A_ reduction, which can be estimated by measuring the kinetics of ChlF relaxation. For calculation of F_r_/F_v_, the area between F_m_ and actual ChlF (F_r1_ or F_r2_) was integrated within a specific time range (t_1_ to t_2_) during ChlF relaxation measurements and normalized to the integral area of F_v_ in that time range (Keller, 2018). The same calculation were made using data from light-adapted measurements. Two time ranges, t_1_ to t_2_, were chosen to catch reoxidation processes with different time constants:

1. t_1_ from 0.8 to 1.47 ms (0.65 ms for calculation of F_r1_)
2. t_2_ from 0.8 to 5.9 ms (5 ms for calculation of F_r2_)

resulting in the efficiency of electron transport 0.65 ms after reduction of Q_A_ (F_r1_/F_v_ in the dark; F_r1_'/F_q_' in the light) and 5 ms after reduction of Q_A_ (F_r2_/F_v_ in the dark; F_r2_'/F_q_' in the light). In contrast to the earlier study of Keller et al. (2019), the time range for t_2_ was chosen to get a more detailed insight into the dynamics of ChlF relaxation. The time ranges of t_1_ and t_2_ correspond to the first and second exponential decay phases after ChlF induction (Vass et al., 1999). These phases are pronounced in dark-adapted samples, in which the photosynthetic apparatus is not activated, but are not visible in light-adapted samples (Keller et al., 2019).

### Spectral Data Processing

Spectral measurements were taken from 400 to 800 nm in 0.46 nm resolution. The detector temperature of the spectrometer was kept between 20 and 35°C. Measurements were acquired in the glasshouse under ambient sunlight every 2 s during the canopy scans. Raw digital numbers from the spectrometer output were averaged to intervals of 2 nm, i.e., to one value per every full even wavelength number between 400 and 800 nm. From every spectra taken during the day, the instrument noise (dark current) measured from spectra at the night before was subtracted. The averaged wavelengths were then used to calculate pseudo spectral indices from the raw digital numbers. The pseudo spectral indices are marked with a "p" at the beginning of the abbreviation, e.g., pseudonormalized difference vegetation index (pNDVI). In addition, reflectance spectra were normalized for incoming irradiance by gray reference spectra (reflecting 50% of the total incoming irradiation). Measurements on the gray reference were carried out in the middle of every measuring round, i.e. once per hour between May 15 and May 18, 2017. The reflectance spectra were then associated with the PPFD value recorded from the environmental station in the same minute and averaged in steps of 10 µmol photons m^−2^ s^−1^. In that way, a look up table for reference spectra was generated for spectra covering a range from 100 to 1,350 µmol photons m^−2^ s^−1^. This look up table was used to correct every spectral measurement according to its associated top of canopy PPFD value using the corresponding reference spectra closest to that PPFD value [Keller (2018) and **Supplementary Figure 1**]. Normalized difference vegetation index (NDVI), alternative NDVI (NDVI_II), green normalized difference vegetation index (GNDVI), MERIS Terrestrial Chlorophyll Index (MTCI), and the PRI were calculated as the following:

1. NDVI = (R750−R706)/(R750+R706) adapted from Frampton et al. (2013)
2. NDVI_II = (R740−R680)/(R740+R680) adapted from Frampton et al. (2013)
3. GNDVI = (R740−R540)/(R740+R540) adapted from Frampton et al. (2013)
4. MTCI = (R754−R710)/(R710+R680) adapted from Dash and Curran (2004)
5. PRI = (R530−R570)/(R530+R570) adapted from Gamon et al. (1992)

with R indicating the used wavelength from the corrected signal. The same wavelengths were used to calculate the pseudo indices using the raw digital signal. A parameter called reflectance was calculated as the sum of the raw signals in all wavelengths between 450 and 800 nm. Every calculated spectral index was then associated to the ChlF measurement taken instantly before.

### Statistical Analysis

Data of ChlF transients were discarded when the signal-to-noise ratio was lower than 50 in the case of maize, rapeseed, and soybean or lower than 100 in barley and wheat. Data were also excluded when F_v_/F_m,_ (respective F_q_'/F_m_') or F_r1_/F_v_ (respective F_r1_'/F_q_') were lower than zero or F_r1_/F_v_ and F_r2_/F_v_ (respective F_r1_'/F_q_' and F_r2_'/F_q_') were higher than 0.35 and 0.8, respectively.

Values from spectral indices were removed when PPFD at that time was <30 µmol photons m^−2^ s^−1^ due to low S/N ratio at low light intensities. Outliers or measurement errors of spectral indices, for example when soil was targeted, were removed when the value was >1.5 times and <1.5 times the second and third quantile of all data collected per species, respectively.

#### Predictive Modeling

Least absolute shrinkage and selection operator (Lasso) regression was performed to identify dependent parameters on phenotypes under fluctuating conditions using *glmnet* package of R program (Friedman et al., 2010). Basic random model equation is:

y = µ + Zu + ε,

where y is a vector of *n* phenotypic values, µ is a common intercept, Z is a *n* x *p* covariate matrix of *p* environmental and reflectance parameters, u is the random effect for every parameter, and ε is a vector of *n* residual. The random effects u are penalized by the ℓ^1^ norm and scaled by a λ value determined by internal cross validation. Parameters for *p* were PPFD, temperature, humidity, vapor pressure deficit (VPD), reflectance, reflectance at 685 nm, GNDVI, NDVI, NDVI_II, PRI, MTCI, age of plants in DAS, measuring week and month, daytime, crop species, and genotype. The predictive models for F_v_/F_m_ (respective F_q_'/F_m_') and F_r2_/F_v_ (respective F_r2_'/F_q_') contained all parameters *p*. Models were fitted on soybean trainings datasets with standardized parameters. Trainings dataset contained either half of all the days measured, the growing season 2016 or 2017. Model predictions were validated on the remaining soybean data. Model accuracy was calculated as Pearson correlation coefficient between predicted and measured values.

#### Linear Modeling

Best predictive parameters for F_v_/F_m_ (respective F_q_'/F_m_') and F_r2_/F_v_ (respective F_r2_'/F_q_') were selected out of the Lasso model for linear modeling in order to quantify the effect of these parameters on photosynthesis. The error term was not further structured (e.g., for correlated errors) since no confidence intervals were calculated. The sum of squares was used to estimate the explained variance of each parameter. To analyze the leaf angle and canopy effect, the factors treatment (fixed *vs.* natural leaf angle) and distance were included in the linear model.

## Results

In order to understand the dynamics of photosynthesis, ChlF response was monitored from the distance under controlled and fluctuating conditions. The photosynthetic response in controlled steady-state conditions was investigated in *Arabidopsis* leaves under different light intensities and temperature levels. Under fluctuating semi-field conditions, four crop species including 28 genotypes were monitored over two growing seasons in order to analyze photosynthetic interactions with the environment. In total, 789,475 measurements were acquired over 138 days using the automated LIFT system.

### Chlorophyll Fluorescence Transients Under Controlled Conditions

Under controlled conditions, ChlF transients of *Arabidopsis* leaves at different temperatures showed differences in the induction phase (**Figure 1A**). As expected, F_q_'/F_m_' responded clearly to increasing light intensities and less pronounced to the different temperatures (**Figure 1B**). In contrast, ChlF relaxation phase and F_r2_'/F_q_' responded highly sensitive to temperatures (**Figure 1C**) but not to light intensities (**Figure 1D**).

### F_v_/F_m_ and F_q_'/F_m_' Under Fluctuating Conditions

**Figure 3:**
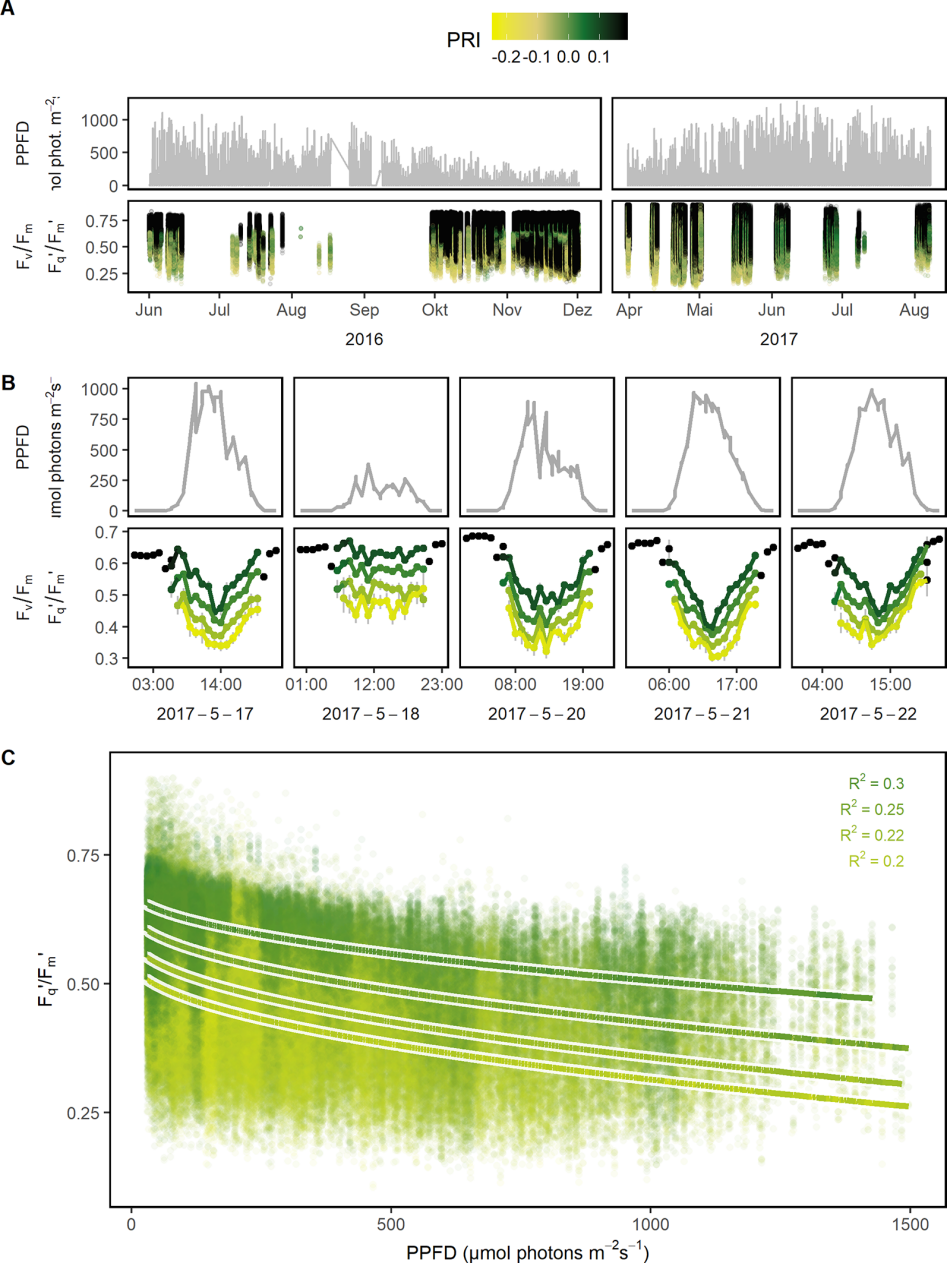
Quantum efficiency of the photosystem II (F_v_/F_m_ in the dark and F_q_'/F_m_' in the light) of barley, maize, soybean, and wheat genotypes was monitored under a fluctuating environment over two seasons in high time resolution. **(A)** Natural fluctuation of photosynthetic photon flux density (PPFD) together with the dynamic F_v_/F_m_ and F_q_'/F_m_' and their associated photochemical reflectance index (PRI) values are shown over the two growing seasons (n = 782,420, acquired from May 2016 to August 2017). **(B)** PPFD and F_v_/F_m_ (respective F_q_'/F_m_') are shown in a subset of diurnal measurements within 1 week to illustrate the strong interaction between the parameters. F_v_/F_m_ and F_q_'/F_m_' were grouped according to photochemical reflectance index (PRI) levels to reveal the strong effect of PRI. Gray error bars show 95% confidence interval (n = 8 to 975 independent measurements averaged per PRI level and hour, 106,433 in total). **(C)** F_q_'/F_m_' was correlated to PPFD, grouped according to PRI levels, and fitted to a linear model depending on PPFD (square root transformed, all measurements with associated PPFD > 25 μmol photons m^−2^ s^−1^ were used, n = 42,434 to 179,152 measurements per PRI group). Chlorophyll fluorescence and spectral data was acquired by an automated light-induced fluorescence transient (LIFT) device scanning over the crop canopy. PPFD was recorded every minute by three stations distributed in the unheated glasshouse and linked to LIFT measurements done in the same minute.

Photosynthetic response under semi-field conditions was monitored in four crop species on canopy level (**Figure 2**). Barley and wheat canopy photosynthesis was monitored for one growing season, maize and soybean for two growing seasons covering a wide range of environmental fluctuations. F_v_/F_m_ (in the dark) and F_q_'/F_m_' (in the light) responded highly dynamically to the fluctuating environmental conditions over the two growing seasons (**Figure 3A**). In a subset of a five diurnal soybean measurements, F_v_/F_m_ and F_q_'/F_m_' showed a clear diurnal pattern following changes in PPFD. F_q_'/F_m_' values were further grouped according to PRI ranges, which are probably related to canopy structure (**Figure 3B**). In a linear model, PPFD explained in total 15.7% of all variance in F_q_'/F_m_' (including 3.7% from a square root term) (**Supplementary Table 3**). PRI explained 21.1%. Further, predicting variables pNDVI_II, measurement date, and pNDVI showed only a minor effect explaining 5.9, 4.3, and 4% of all variance in F_q_'/F_m_,' respectively. Temperature (accounting only for 0.3% of the variance), humidity, crop species, and genotype had no major effect on F_q_'/F_m_.' The interaction of PRI with F_q_'/F_m_' was rather stable and independent of PPFD (**Figure 3C**). The unexplained variance was 39.5%. In summary, F_v_/F_m_ and F_q_'/F_m_' under fluctuating conditions were mainly dependent on PPFD and reflectance indices but little affected by temperature or measurement date.

### F_r2_/F_v_ and F_r2_'/F_q_' Under Fluctuating Conditions

**Figure 4:**
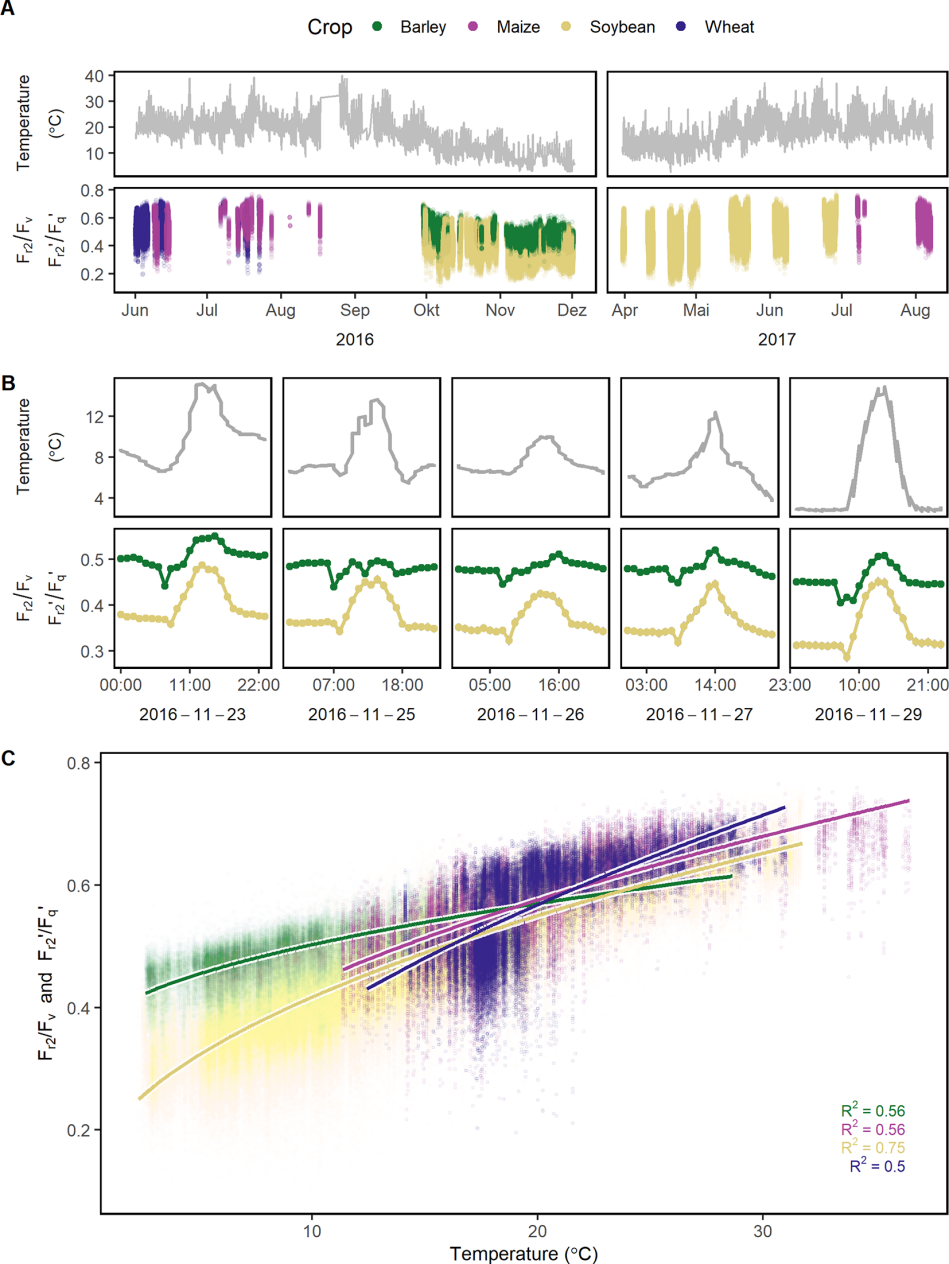
Reoxidation efficiency 5 ms after primary quinone (Q_A_) reduction (F_r2_/F_v_ in the dark and F_r2_'/F_q_' in the light) of barley, maize, soybean, and wheat genotypes was monitored under a fluctuating environment over two seasons in high time resolution. **(A)** Temperature fluctuation together with the dynamic F_r2_/F_v_ and F_r2_'/F_q_' of the four crop species is shown over the two growing seasons (n = 778,224, acquired from May 2016 to August 2017). **(B)** Temperature and F_r2_/F_v_ (respective F_r2_'/F_q_') are shown in a subset of diurnal measurements within 1 week to illustrate the strong interaction between the parameters. F_r2_/F_v_ and F_r2_'/F_q_' were grouped according to crop species to reveal the strong effect of the different species. Gray error bars show 95% confidence interval (n = 58 to 171 independent measurements averaged per crop species and hour, in total = 30,704). **(C)** F_r2_/F_v_ and F_r2_'/F_q_' were correlated to temperature and fitted to a linear model depending on temperature (temperature was square root transformed, all measurements were used, n = 33,902 to 604,857 measurements per crop species). Chlorophyll fluorescence data was acquired by an automated light-induced fluorescence transient (LIFT) device scanning over the crop canopy. Temperature was recorded every minute by three stations distributed in the unheated glasshouse and linked to LIFT measurements done in the same minute.

F_r_/F_v_ (in the dark) and F_r_'/F_q_' (in the light) describe the oxidation kinetics of Q_A_^−^. In order to catch different steps in the electron transport, two time constants (t_1_ = 0.65 ms and t_2_ = 5 ms) were considered in this study. F_r1_/F_v_ and F_r1_'/F_q_' were highly correlated to F_r2_/F_v_ and F_r2_'/F_q_' in all four crops whereas the ratio depended on the measurement time (**Supplementary Figure 3**). Therefore in the following, this study is focused only on F_r2_/F_v_ and F_r2_'/F_q_.' The parameter responded highly dynamically to the fluctuating environment (**Figure 4A**). In a data subset, F_r2_/F_v_ and F_r2_'/F_q_' of barley and soybean showed a clear distinct diurnal pattern dependent on temperature and species (**Figure 4B**). In a linear model for F_r2_/F_v_ and F_r2_'/F_q_,' temperature alone explained over 67% of all variance in F_r2_/F_v_ and F_r2_'/F_q_' (**Table 1**). The different months of the season and crop species accounted for 5.1 and 5% of the variance, respectively. Interestingly, winter barley had higher F_r2_/F_v_ and F_r2_'/F_q_' values than soybean in cold temperature but lower values in warm temperature (**Figure 4C**). In contrast to F_q_'/F_m_,' PRI and pNDVI did not contribute significantly to variation in the data. The unexplained variance was 21%. The two monitored ChlF parameters, F_v_/F_m_ (respective F_q_'/F_m_') and F_r2_/F_v_ (respective F_r2_'/F_q_'), were independent form each other and changed their relation according to the time of the day (**Supplementary Figure 4**). In contrast to F_v_/F_m_ and F_q_'/F_m_,' the parameters F_r2_/F_v_ and F_r2_'/F_q_' were highly dependent on temperature.

**Table 1:** Reoxidation efficiency 5 ms after primary quinone (Q_A_) reduction (F_r2_/F_v_ in the dark and F_r2_'/F_q_' in the light) measured in four crop species over two seasons in an unheated glasshouse was analyzed using a linear model (n = 760,874 measurements). Depending factors were temperature (including a square root term), time point of the measurement (month, date, and hour), crop species, genotype, plot, and days after sowing (DAS). Descriptors of the linear models are degree of freedom (Df), sum of squares, mean of squares, ratio of mean squares, and mean squares error (F value) and the explained sum of squares per factor (explained variance) in percentage.

	Df	Sum squares	Mean squares	F value	Explained variance (%)
Temperature	1	4,858.15	4,858.15	2,942,365	67.2
Residuals	760,640	1,255.9	0	NA	17.4
Month	12	366.97	30.58	18,521.5	5.1
Crop	2	361.88	180.94	109,587.8	5
Hour	23	206.15	8.96	5,428.6	2.9
Date	123	78.87	0.64	388.3	1.1
Genotype	24	53.54	2.23	1,351.2	0.7
Plot	46	21.07	0.46	277.4	0.3
DAS	1	17.22	17.22	10,431.2	0.2
Temperature^0.5^	1	12.09	12.09	7,322.7	0.2

### Detection of Cold Tolerance in Soybean Genotypes

**Figure 5:**
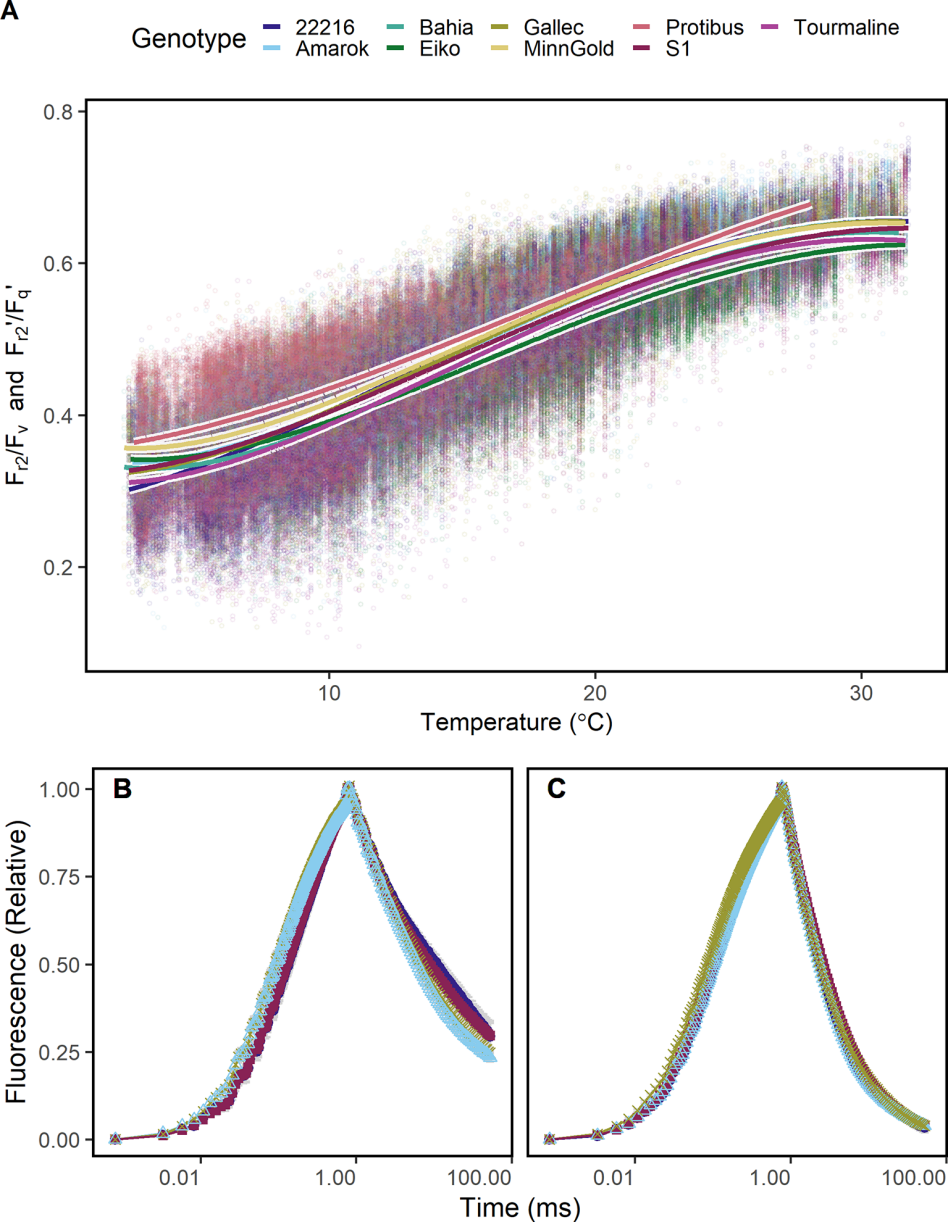
Chlorophyll fluorescence transients were measured in soybean canopies of different genotypes over two seasons under fluctuating conditions. **(A)** Efficiency of electron transport 5 ms after reduction of primary quinone (Q_A_) (F_r2_/F_v_ in the dark, F_r2_'/F_q_' in the light) of soybean genotypes differing in cold tolerance are shown. F_r2_/F_v_ and F_r2_'/F_q_' were fitted to a linear model depending on temperature (including a square root and a squared temperature term). **(B)** A contrasting subset of chlorophyll fluorescence transients of genotypes 22216, Amarok, Gallec, and S1 at 4°C and **(C)** 20°C is shown. F_r2_/F_v_ and F_r2_'/F_q_' calculated from these data are shown in **Supplementary Figure 5**. For each temperature level, measurements were selected from October and November 2016 which had data recorded at the indicated temperature ( ± 0.5°C) between noon and dawn. Error bars show 95% confidence interval of the mean (n = 33 to 161 measurements). Genotype Amarok and Gallec are breed for cold tolerance. Light-induced fluorescence transient (LIFT) method was used with fast repetition rate flash from about 1 m distance scanning over the crop canopy. Temperature was recorded every minute by three stations distributed in the unheated glasshouse and linked to LIFT measurements done in the same minute.

Since F_r2_/F_v_ and F_r2_'/F_q_' may be a proxy for temperature dependent limitations in electron transport, it was tested whether these ChlF parameters detect cold tolerance. The response of F_r2_/F_v_ and F_r2_'/F_q_' to temperature differed between the genotypes especially at low and high temperature (**Figure 5A**). Genotype Protibus had no data around 30°C because it was measured only in 2016. Data acquired at 5°C revealed faster ChlF relaxation in cold-tolerant genotype Amarok and Gallec compared to 22216 or S1 (**Figure 5B**). At 5°C, F_r2_/F_v_ and F_r2_'/F_q_' between these four genotypes differed significantly whereas F_v_/F_m_ showed no difference (**Supplementary Figure 5**). In contrast, these genotypes showed no clear difference in the ChlF relaxation at 20°C (**Figure 5C**). In summary, F_r2_/F_v_ and F_r2_'/F_q_' allowed to monitor temperature tolerance of the photosynthesis in different genotypes.

### Genotype x Environment Interaction and Modeling in Soybean Genotypes

**Figure 6:**
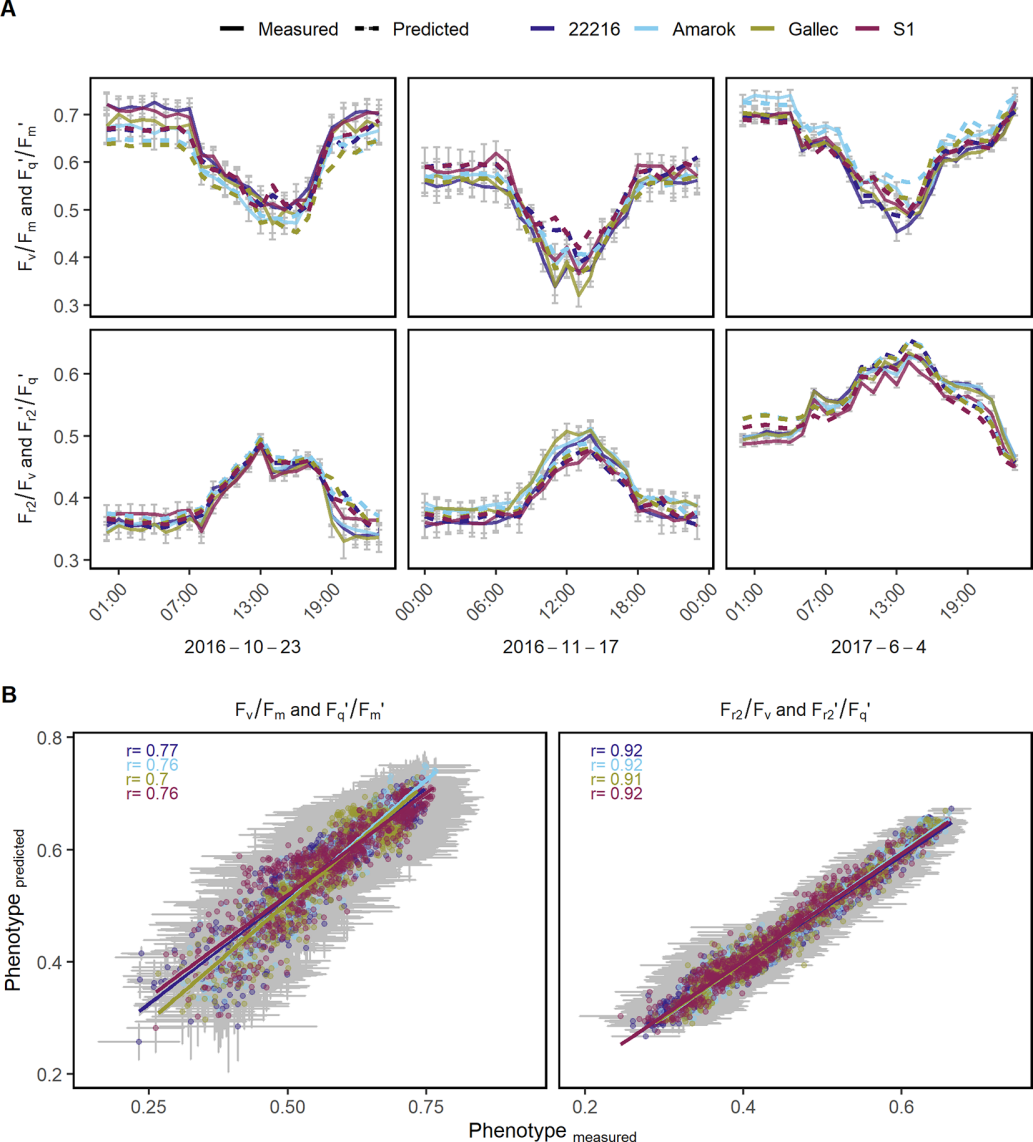
Chlorophyll fluorescence parameters were used to model genotype x environment interactions of different soybean genotypes. **(A)** Quantum efficiency of the photosystem II (F_v_/F_m_ in the dark and F_q_'/F_m_' in the light) and the efficiency of electron transport 5 ms after reduction of primary quinone (Q_A_) (F_r2_/F_v_ in the dark, F_r2_'/F_q_' in the light) at three random dates with measured (full line) and modeled (dashed line) values are shown. **(B)** Predicted and measured values for both photosynthetic parameters were correlated to each other in order to assess the model accuracy. Measurements of 48 days were used to train the model using Lasso regression (n = 284,662). Photosynthetic performance over the remaining 47 days was predicted and validated (n = 295,885). Predicted and measured values were averaged per genotype, day and hour. Error bars show the 95% confidence interval. The λ value derived through internal cross-validation on the training set was 1.14285 x 10^−5^ and 3.626669 x 10^−5^ for F_v_/F_m_ and F_r2_/F_v_, respectively. For the Lasso model coefficients see **Supplementary Table 4**.

The genotype specific response of the photosynthetic parameters to natural fluctuation under semi-field conditions was modeled in order to estimate genotype x environment interactions over full seasons. F_v_/F_m_ and F_q_'/F_m_' as well as F_r2_/F_v_ and F_r2_'/F_q_' were modeled based on environmental data and reflectance indices. Photosynthetic performance of 69 days were predicted in high time resolution using the remaining 69 days to train the model (in total 580,547 measurements). In a subset of three dates, the model based estimates of the photosynthetic parameters are shown together with the measured values as validation (**Figure 6A**). The prediction accuracies, expressed as the Pearson correlation coefficient of estimated and measured values, ranged between 0.7 and 0.92 for the different genotypes in F_q_'/F_m_' (and F_v_/F_m_) and F_r2_'/F_q_' (and F_r2_/F_v_), respectively (**Figure 6B**). The model coefficients are shown in **Supplementary Table 4** and **Supplementary Datasheet 2**respectively. Furthermore, we modeled genotype x environment interactions over an entire season based on the other measured season. These prediction accuracies ranged between 0.44 and 0.84 for the different genotypes in F_q_'/F_m_' (and F_v_/F_m_) and F_r2_'/F_q_' (and F_r2_/F_v_), respectively (**Supplementary Figure 6**). In summary, the modeling of genotype x environment interactions allowed the estimation of the photosynthetic performance also at days or entire seasons which had no measurements available.

### Influence of Canopy Structure, Plant Height, and Leaf Angle

In order to estimate the variance introduced by canopy structure, soybean leaves were fixed perpendicular to the LIFT lens. At noon and night, LIFT signal was not significantly affected by changes of the measuring distance within 150 mm (**Supplementary Figure 7**). The effect of the measuring distance ranging from 750 to 1,200 mm on F_v_/F_m_ and F_r2_/F_v_ accounted for about 1.4 and 19.3% of the variance during night measurements, respectively (**Supplementary Table 5**). In contrast, leaf angle (or canopy structure) affected F_v_/F_m_ more strongly (23.6% of explained variance) than F_r2_/F_v_ (2.2% of explained variance). At noon, the explained variance of F_q_'/F_m_' and F_r2_'/F_q_' for leaf angle was not bigger than 8% (**Supplementary Table 6**). Regarding PRI, leaf angle accounted for 22.7% of explained variance representing canopy structure. In summary, photosynthesis, specifically regulation of F_q_'/F_m_' and F_r2_'/F_q_,' in response to a fluctuating environment was quantified and related to environmental factors and canopy structure.

## Discussion

Based on diurnal and seasonal ChlF data from different crop species and genotypes, a unique dataset was collected to identify, quantify, and model photosynthesis x environmental interactions under semi-field conditions. F_v_/F_m_ and F_q_'/F_m_' correlated with PPFD but not significantly with temperature. Therefore, screening for this trait detects light-use efficient genotypes at the level of PSII charge separation but misses the photosynthetic response to temperature. In contrast, F_r2_/F_v_ and F_r2_'/F_q_' provided information about genotype specific differences in acclimation to temperature. In general, the responses to temperature and PPFD from lab conditions in *Arabidopsis* to fluctuating environmental conditions in the four different crop species were consistent in their trends but differed in the complexity of the interactions.

### Temperature Tolerance of Electron Transport

For the first time, F_r2_/F_v_ and F_r2_'/F_q_' were quantified under fluctuating semi-field conditions and their interactions with the environment was analyzed. F_r2_/F_v_ and F_r2_'/F_q_' were highly dependent on temperature pointing toward limitations of electron transport in the cold. Under cold conditions, electron transport depends on the membrane fluidity, which is maintained by the fatty acid composition (Upchurch, 2008; Liu et al., 2013). Electron transport between PSII and PSI acclimates to different growth temperatures which was shown by measurements of Q_A_^−^ reoxidation and PSI primary donor (P700) reduction (Yamasaki et al., 2002). Exposure to cold of non-acclimated plants resulted in a severe loss of thylakoid membrane function and decreased NPQ capacity in rice plants (Suzuki et al., 2011). The efficiency to reduce the electron transport chain downstream of Q_A_ was reported to be increased after six nights of dark chilling compared to the control (Van Heerden et al., 2003). In general, exposure to cold of non-acclimated plants resulted in a severe loss of thylakoid membrane function, decreased reoxidation efficiency of Q_A_^−^, and limited electron transport and carboxylation rate (Yamori et al., 2008; Suzuki et al., 2011; Krüger et al., 2014). This probably explains the close relationship of F_r2_/F_v_ and F_r2_'/F_q_' to temperature which we found under controlled (**Figure 1D**) and fluctuating conditions (**Figure 4C**). F_r2_'/F_q_' seemed to reflect the capacity of electron transport from Q_A_ through PQ pool toward cytochrome *b*_6_ *f* and PSI complex. In the light-adapted state, F_r1_'/F_q_' and F_r2_'/F_q_' were highly correlated indicating a continuous electron transport (**Supplementary Figure 3**). In agreement, a gradual decrease of the ChlF relaxation curve was observed in light-adapted leaves under controlled conditions (Keller et al., 2019). The changes in the ratio between F_r1_'/F_q_' and F_r2_'/F_q_' according to the time of the day were probably related to the diurnal temperature pattern. Ratio changes in the morning seemed to represent the acclimation of the photosynthetic machinery to light visible as temporary decrease in F_r2_'/F_q_' (**Figure 4B**). This transition was also observed under lab conditions upon illumination of dark-adapted leaves (Keller et al., 2019). In photosynthetic models, the response to temperature is commonly included as a constant (Bernacchi et al., 2001; Yamori et al., 2014). Under fluctuating environmental conditions, F_r2_/F_v_ and F_r2_'/F_q_' could improve these models exchanging the constant by a genotypic specific variable responding to temperature at the level of electron transport.

The detected temperature tolerance of photosynthetic electron transport varied between species and genotypes (**Figure 5A**). In general, the minimum temperature required for growth is 5°C (Körner, 2016). The cold tolerant genotype Amarok showed higher F_r2_/F_v_ (respective F_r2_'/F_q_') than the cold sensitive genotypes 22216 and S1 at low temperature. This tolerance of electron transport to low temperature may be related to adjusted membrane composition or general limitations at CO_2_ fixation (Liu et al., 2013; Yamori et al., 2014; Pignon et al., 2019). The remaining cold tolerant genotypes, Tourmaline and Gallec, showed intermediate response indicating different cold tolerance mechanisms in soybean (Gass et al., 1996; Yamori et al., 2010). The potential to detect cold tolerance *via* the analysis of electron transport kinetics was also demonstrated earlier under lab conditions (Strauss et al., 2006; Krüger et al., 2014). We conclude that F_r2_/F_v_ and F_r2_'/F_q_' represent efficiency of electron transport beyond Q_A_ reflecting membrane fluidity and composition, and therefore contributes to the temperature tolerance at a given temperature.

### Photosynthetic Interactions With Light Intensity and Canopy Architecture

In steady-state conditions, F_q_'/F_m_' is tightly linked to electron transport and CO_2_ assimilation (Genty et al., 1989; Niyogi et al., 1998; Von Caemmerer, 2000). Under natural conditions, F_v_/F_m_ and F_q_'/F_m_' follow a diurnal pattern (Adams and Demmig-Adams, 1995; Ribeiro et al., 2004; Pieruschka et al., 2008; Moura dos Santos et al., 2013; Ruiz-Vera et al., 2015). Similar results, but with higher spatio-temporal resolution over the whole seasons, were presented in this study (**Figure 3A**). The response of F_q_'/F_m_' to light measured under fluctuating semi-field conditions was almost linear (**Figure 3C** and **Supplementary Table 1**). It did not fit the response measured under controlled steady-state conditions (**Figure 1B**). In agreement, the curvature factor and the light saturation point were reported to be reduced under natural light conditions compared to control conditions probably caused by higher NPQ levels (Rascher et al., 2000; Jia et al., 2013; Meacham et al., 2017). In relation to that, the response of F_q_'/F_m_' to temperature was negligible under fluctuating conditions in contrast to lab conditions (**Supplementary Table 1**). The decrease of F_q_'/F_m_' under cold stress was associated with the inhibition of PSII reaction centers and their repair mechanism (Murata et al., 2007). Cold acclimation resulted in slower decrease of F_v_/F_m_ after exposure to 4°C in control leaves compared to cold-hardened leaves (Streb et al., 1999). This could explain the small effect of temperature on F_q_'/F_m_' under fluctuating conditions allowing cold acclimation compared to lab conditions. Comparing fluctuating with controlled conditions, we conclude that the response of F_q_'/F_m_ to light intensity was slightly modified by higher NPQ levels while the response to temperature was minimized by cold acclimation.

Besides PPFD, F_q_'/F_m_' on canopy level was related to PRI in all four species examined in this study (**Figure 3C**). PRI is mainly linked to the xanthophyll cycle and therefore sensitive to NPQ changes and various other stress responses (Prasad et al., 2006; Garbulsky et al., 2011; Zhang et al., 2016). It explains the close relationship of PRI and F_q_'/F_m_' described in a recent meta-analysis (Sukhova and Sukhov, 2018). However, scans of natural crop canopy showed additionally a high variability in NDVI (e.g., **Supplementary Figure 2**). The NDVI is known to correlate highly with vegetation productivity, hence, we would not expect a high variability within one plot (Gamon et al., 1992; Ji and Peters, 2003). This variability could be explained with the observation that PRI as well as NDVI vary with canopy structure (Barton and North, 2001; Rascher et al., 2015; Cordon et al., 2016). Canopy structure affects physiological processes directly, e.g., the leaf angle distribution affects the light penetration into the canopy leading to variation in F_q_'/F_m_' and NPQ. In addition, F_q_'/F_m_' values differed in the upper compared to the lower canopy and were affected by steep leaf angles (Rascher and Pieruschka, 2008; Wyber et al., 2018). Our data support this conclusion: variability in F_q_'/F_m_,' NPQ, and PRI were higher in leaves with natural orientation than in leaves with a fixed leaf angle (**Supplementary Figure 7**). The leaf angle explained more of the variation in PRI than in F_q_'/F_m_' indicating an additional influence of canopy structure to PRI (**Supplementary Table 6**). Similarly, the correlations between PRI and F_q_'/F_m_' decreased when measured on canopy compared to leaf level (Sukhova and Sukhov, 2018). These findings indicate a combination of NPQ level and canopy structure expressed in F_q_'/F_m_' and PRI. The variability in F_q_'/F_m_' on canopy level describes plant performance in the field more realistic than measurements on selected leaves or leaf segments (Evans, 2013; Niinemets et al., 2015). In conclusion, the variability of F_v_/F_m_ and F_q_'/F_m_' combines physiological and structural canopy information without the requirement to select leaves for measurement under steady-state conditions.

### Prediction of Photosynthesis in a Fluctuating Environment

Photosynthetic genotype x environment interactions were modeled over the entire growing season based on environmental parameters and training data from another season (**Supplementary Figure 6**). The accurate prediction across seasons (Pearson correlation coefficient between 0.44 and 0.84) indicated that our modeling is valid for a wide range of environmental conditions confirming the identified predictive parameters. Based on these models, the total amount of electron transported through a season for a specific genotype is possible to estimate without having measurement data from that season. This has potential application in crop growth models to increase the prediction of plant performance in untested environments (van Eeuwijk et al., 2019; Voss-Fels et al., 2019). The LIFT method is directly applicable in high-throughput field phenotyping requiring about 30 s to scan 1 m plot. A few diurnal measurements over the season seem to be sufficient to model the full photosynthetic response. Different development stages were probably represented in our models *via* seasonal changes of reflectance indices such as NDVI (Condorelli et al., 2018). In the soybean data of 2017, development stage represented by DAS was correlated to NDVI with a Pearson correlation coefficient of 0.63 (data not shown). Further research is needed to increase the model prediction accuracies and to gain more detailed knowledge about driving factors of photosynthesis in the field. Based on the detected environmental interactions, the modeling and estimation of photosynthetic performance at the genotype level over entire growing seasons is possible.

## Conclusions

Diurnal and seasonal fluctuation of photosynthesis at canopy level was successfully quantified using ChlF measurements in high-throughput. F_v_/F_m_ and F_q_'/F_m_ and the newly established ChlF parameters F_r2_/F_v_ and F_r2_'/F_q_' were able to detect photosynthetic acclimation under fluctuating semi-field conditions. F_q_'/F_m_ provided the quantum efficiency at the level of PSII and was mainly determined by PPFD. In contrast, F_r2_'/F_q_' was rather independent of PPFD and reflected efficiency of electron transport beyond Q_A_. F_r2_/F_v_ and F_r2_'/F_q_' showed a high sensitivity to temperature identifying electron transport limitations at low temperature when F_v_/F_m_ was not affected. The automated scans allowed a high spatio-temporal resolution of the data. It enabled the analysis of several genotypes regarding not only means under steady-state conditions but also their dynamic interaction with environmental factors. Autonomous monitoring of photosynthesis x environment interactions under natural conditions as well as their predictions over entire growing seasons has great potential in plant physiology and breeding applications.

## Data Availability Statement

The datasets generated and analyzed for this study can be found under the DOI 10.5281/zenodo.3260253.

## Author Contributions

BK, SM, OM, UR, and RP designed the study. BK, AS, TK, and OM carried out the experiments, AS, OM, RP, SM, and UR contributed materials and advice. BK and SM wrote the manuscript with contributions from TK, UR, and OM.

## Funding

German-Plant-Phenotyping Network funded by the German Federal Ministry of Education and Research (project identification number: 031A053).

## Conflict of Interest

The authors declare that the research was conducted in the absence of any commercial or financial relationships that could be construed as a potential conflict of interest.
