# Supplementary material for: Genotype Specific Photosynthesis x Environment Interactions Captured by Automated Fluorescence Canopy Scans Over Two Fluctuating Growing Seasons
Source: Front Plant Sci. 2019 Nov 20;10:1482. doi: 10.3389/fpls.2019.01482 (PMC6962999; doi:10.3389/fpls.2019.01482)
Supplement: Supplementary file 1 [file DataSheet_1.docx]

Supplementary Material

# Supplementary Figures


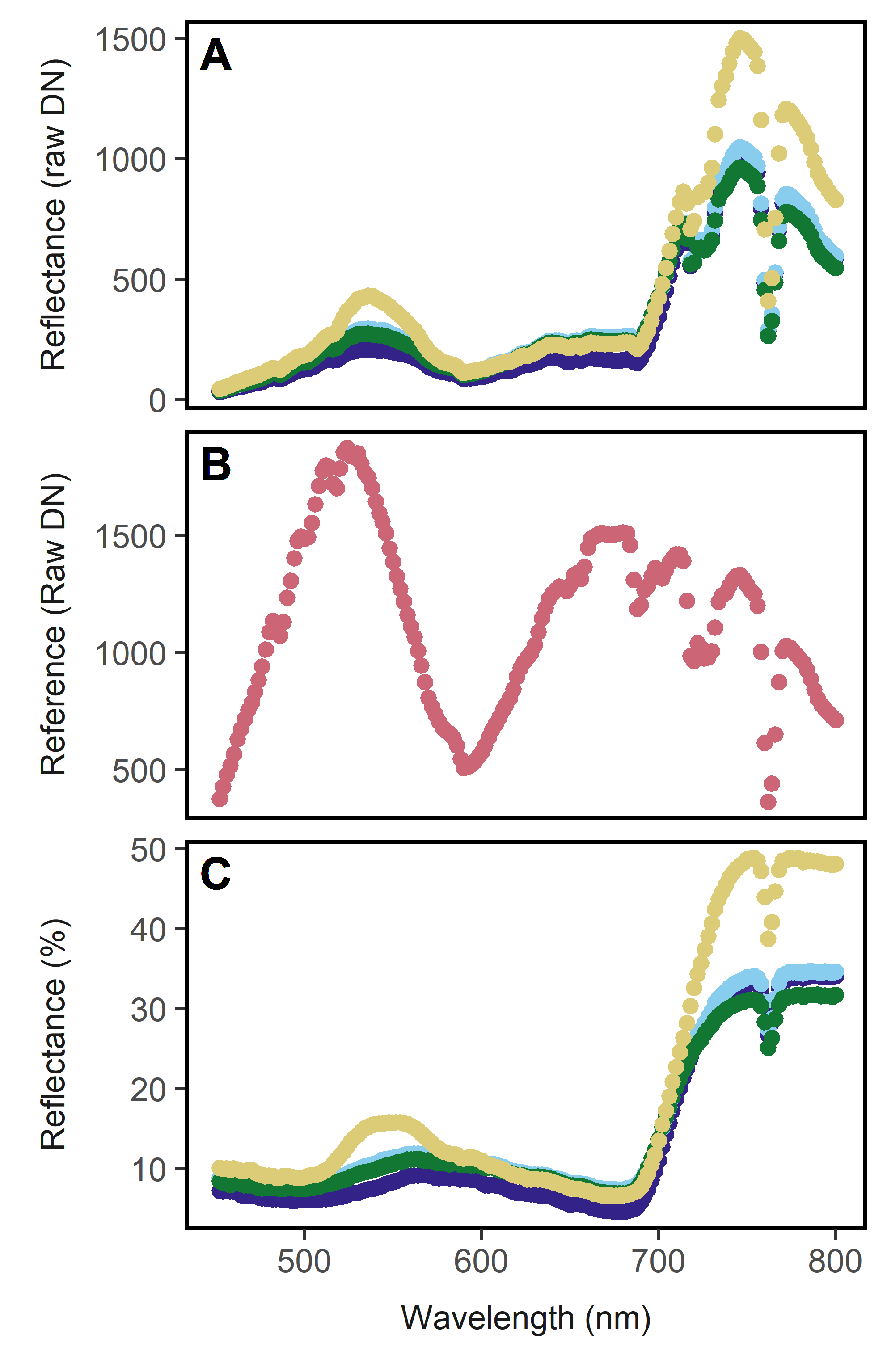


Supplementary Figure 1

Four examples of spectral measurements taken at noon over one soybean plot showing uncorrected reflectance in raw digital numbers (A), the associated grey reference at the same light intensity (B) and the normalized reflectance spectra (C). Light-induced fluorescence transient (LIFT) device with built in spectrometer was used scanning over the crop canopy. The spectral integration time was 1,790 ms.


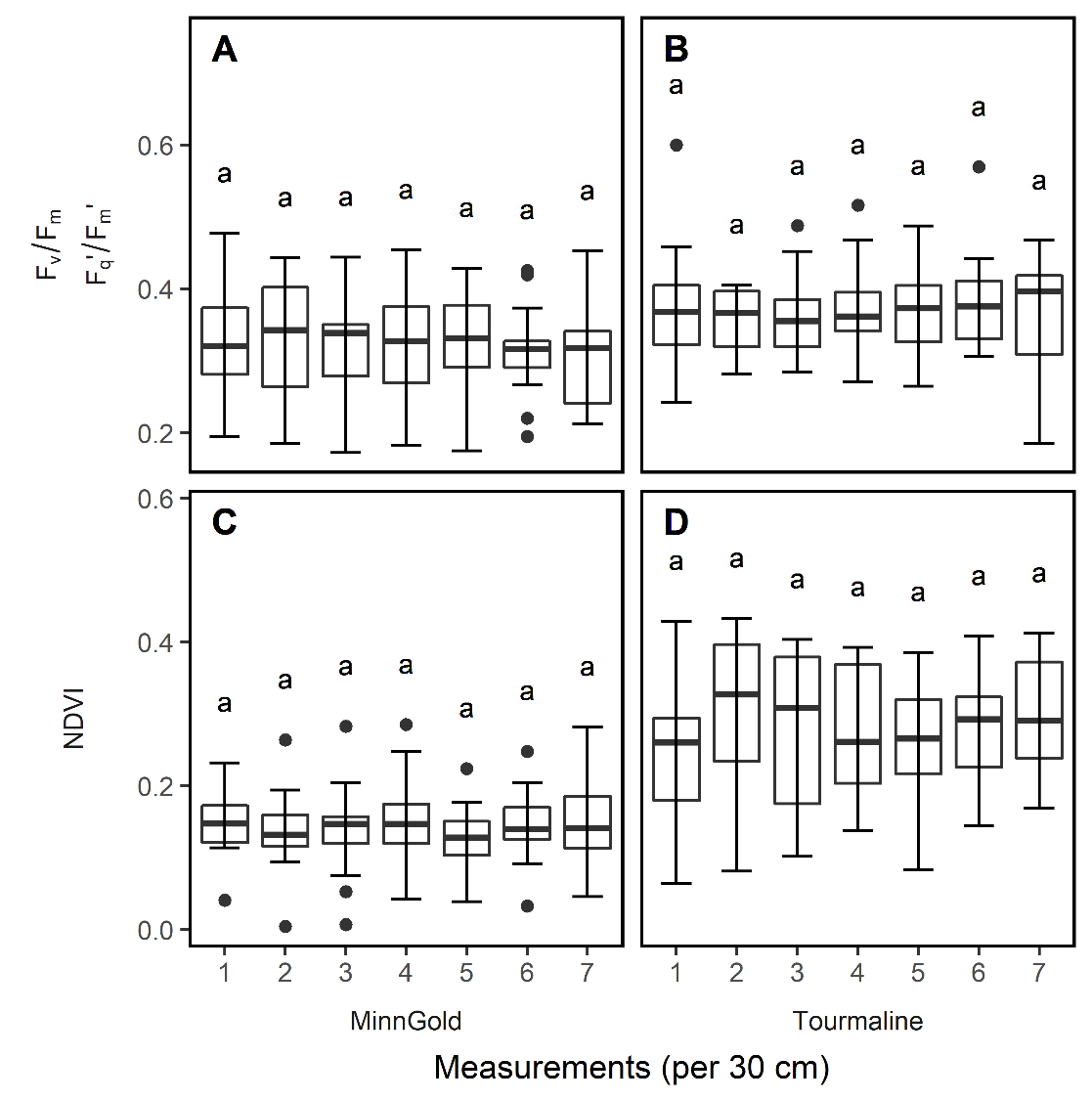


Supplementary Figure 2

Boxplot of quantum efficiency of the photosystem II (F_v_/F_m_ in the dark and F_q_’/F_m_’ in the light), and normalized difference vegetation index (NDVI) for genotype MinnGold (A, C) and Tourmaline (B, D) for the approximate seven measurements per 300 mm scanning line are shown. Measurements were taken at noon of each four plots (16 steps). Box represents inter-quartile range, bold horizontal bar the median, the discontinuous lines the upper and lower quartile, and outlier datapoints (>1.5 × inter-quartile range) are depicted by a point. Means with different letters differ significantly using one way ANOVA followed by Tukey’s multiple comparisons of means.


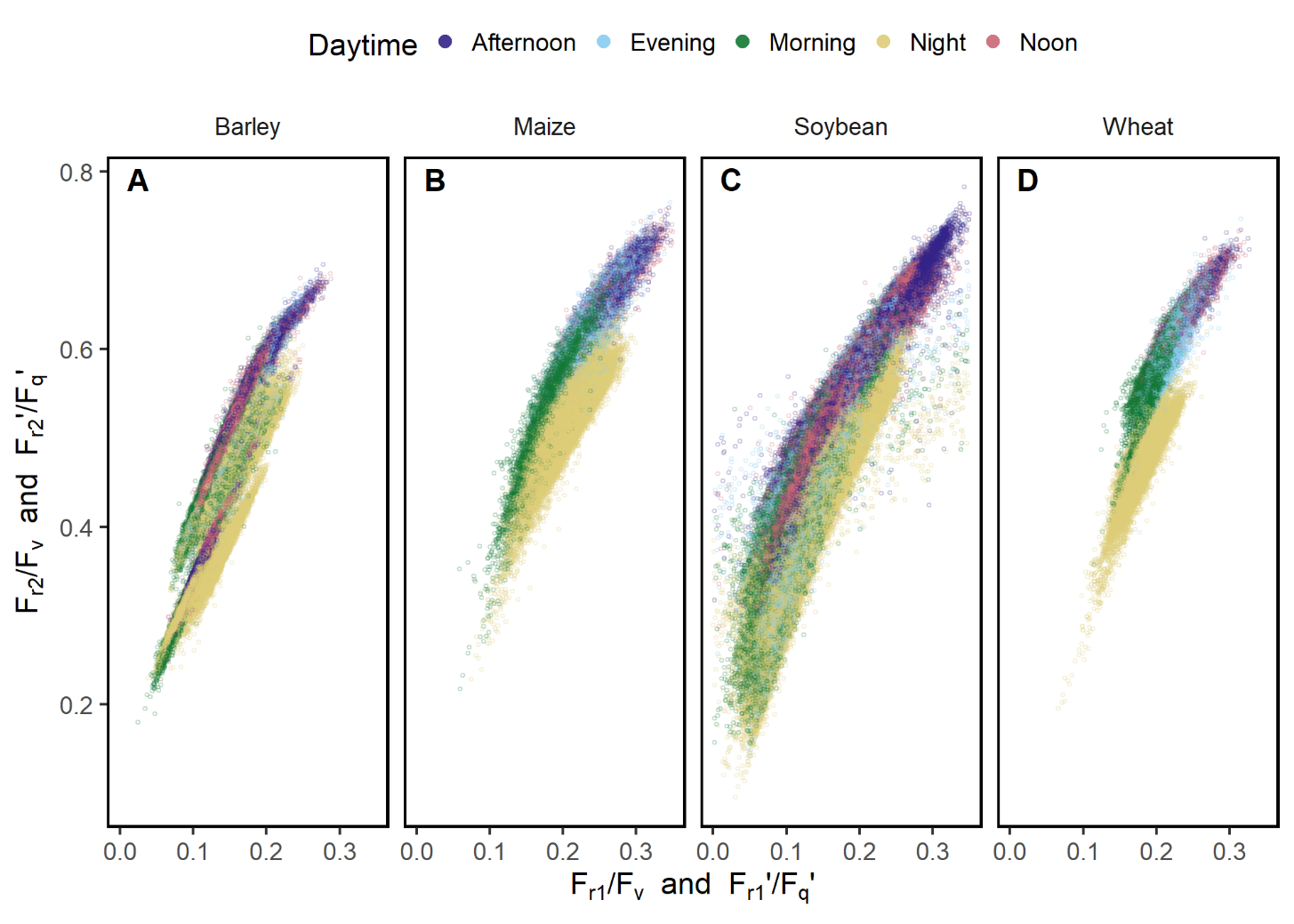


Supplementary Figure 3

Reoxidation efficiency 0.65 ms after primary quinone (Q_A_) reduction (F_r1_/F_v_ in the dark and F_r1_’/F_q_’ in the light) correlated to reoxidation efficiency 5 ms after primary quinone (Q_A_) reduction (F_r2_/F_v_ in the dark and F_r2_’/F_q_’ in the light) in barley (A), maize (B), soybean (C) and wheat (D) grouped after time of the day. Chlorophyll fluorescence data were acquired by a light-induced fluorescence transient (LIFT) device scanning the crop canopy from an automated moving platform (n= 784,534)


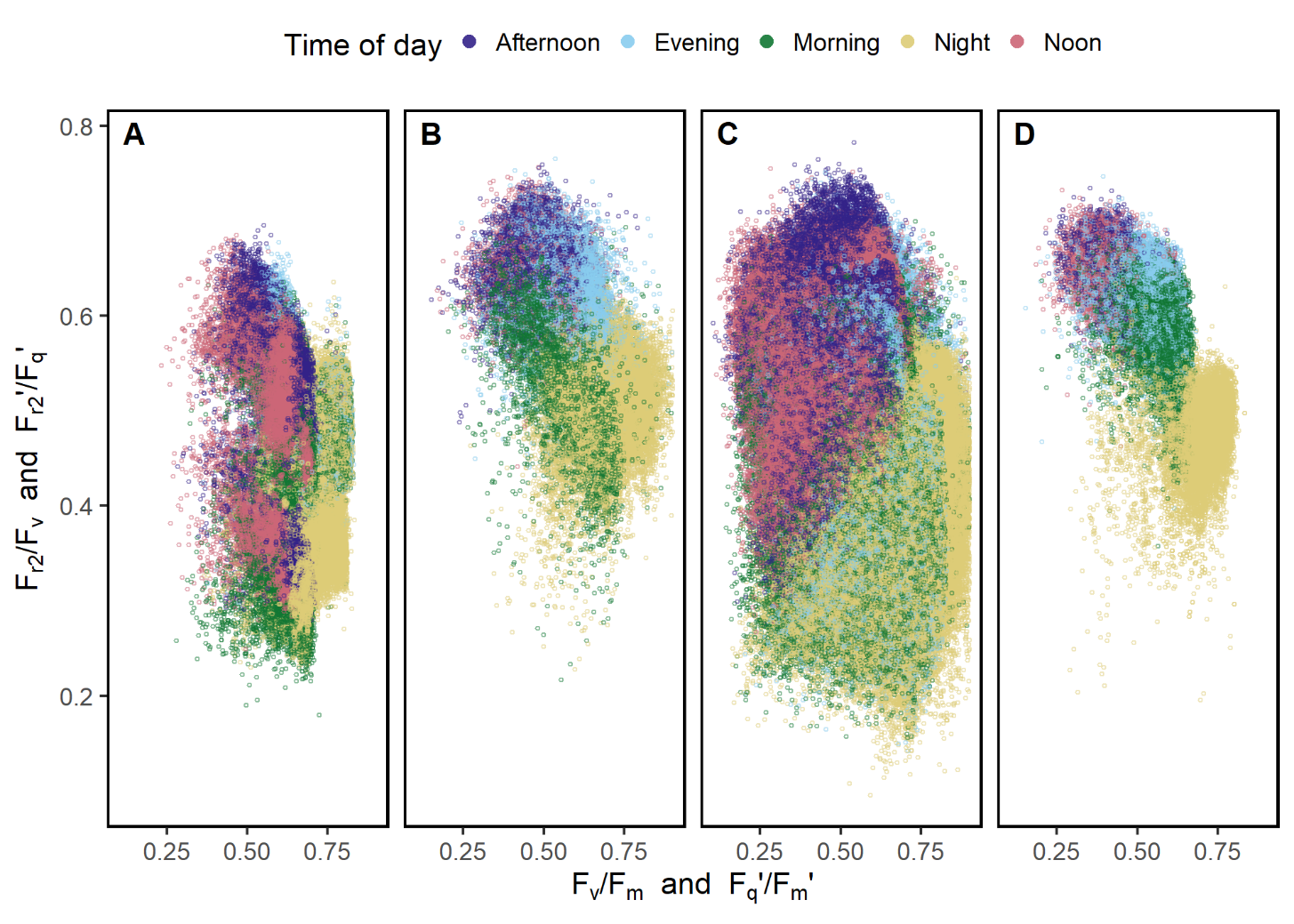


Supplementary Figure 4

Operating efficiency of the photosystem II (F_v_/F_m_ in the dark and F_q_’/F_m_’ in the light) correlated to reoxidation efficiency 5 ms after primary quinone (Q_A_) reduction (F_r2_/F_v_ in the dark and F_r2_’/F_q_’ in the light) in barley (A), maize (B), soybean (C) and wheat (D) grouped after time of the day. Chlorophyll fluorescence data were acquired by a light-induced fluorescence transient (LIFT) device scanning the crop canopy from an automated moving platform (n= 784,534)


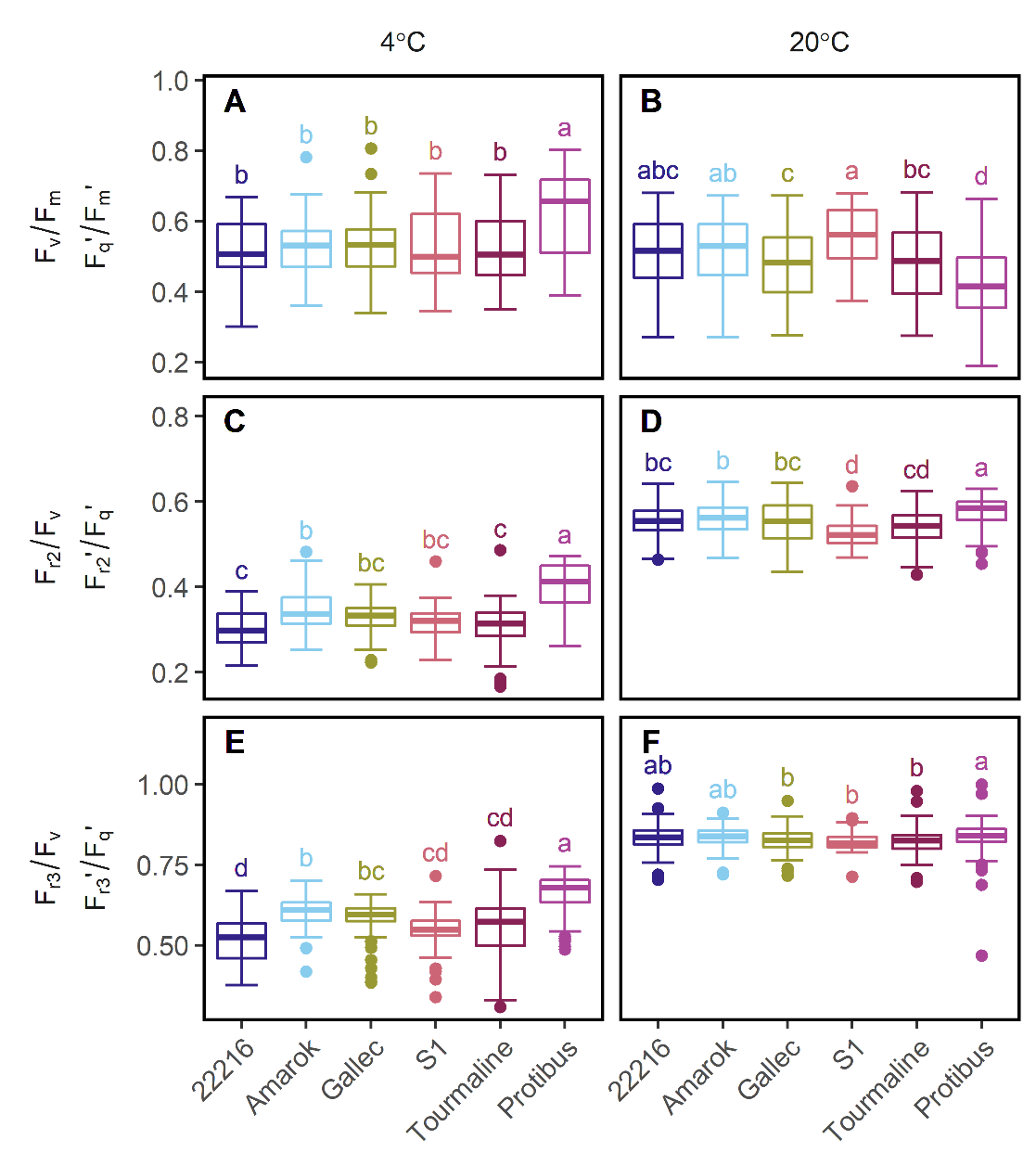


Supplementary Figure 5

Boxplot of operating efficiency of the photosystem II (F_v_/F_m_ in the dark and F_q_’/F_m_’ in the light, A, B), reoxidation efficiency 5 ms after primary quinone (Q_A_) reduction (F_r2_/F_v_ in the dark and F_r2_’/F_q_’ in the light, C, D) and reoxidation efficiency 30 ms after Q_A_ reduction (F_r3_/F_v_ in the dark and F_r3_’/F_q_’ in the light, E, F) are shown of soybean genotypes measured at 5°C and 20°C. Light-induced fluorescence transient (LIFT) method was used with fast repetition rate flash from about 1 m distance scanning over the crop canopy. Temperature was recorded every minute by three stations distributed in the unheated glasshouse and associated to LIFT measurements done in the same minute. For each temperature, three measurement days were selected (n=33 to 202 measurements per genotype and temperature). Box represents inter-quartile range, bold horizontal bar the median, the discontinuous lines the upper and lower quartile, and outlier data points (>1.5 × inter-quartile range) are depicted by a point. Means with different letters differ significantly (p<0.05) using one way ANOVA followed by Tukey’s multiple comparisons of means


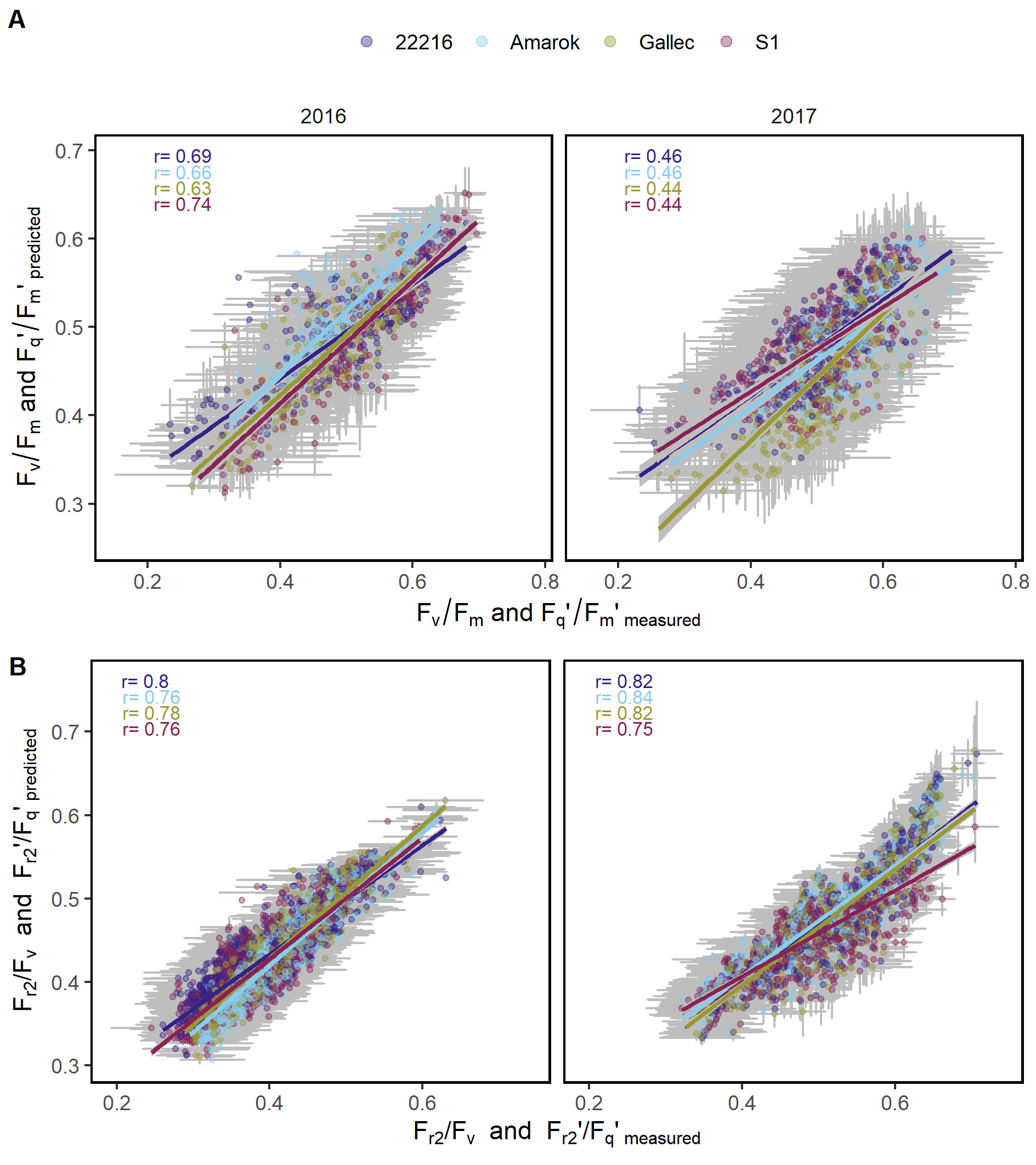


Supplementary Figure 6

Chlorophyll fluorescence parameters predicted for an entire growing season for different soybean genotypes. Operating efficiency of the photosystem II (F_v_/F_m_ in the dark and F_q_’/F_m_’ in the light) (A) and reoxidation efficiency 5 ms after primary quinone (Q_A_) reduction (F_r2_/F_v_ in the dark and F_r2_’/F_q_’ in the light) (B). The model was built on environmental and spectral parameters using ridge regression and data of one year to predict the other one. Predicted and measured values were averaged per genotype, day and hour and correlated to each other in order to assess the model accuracy.


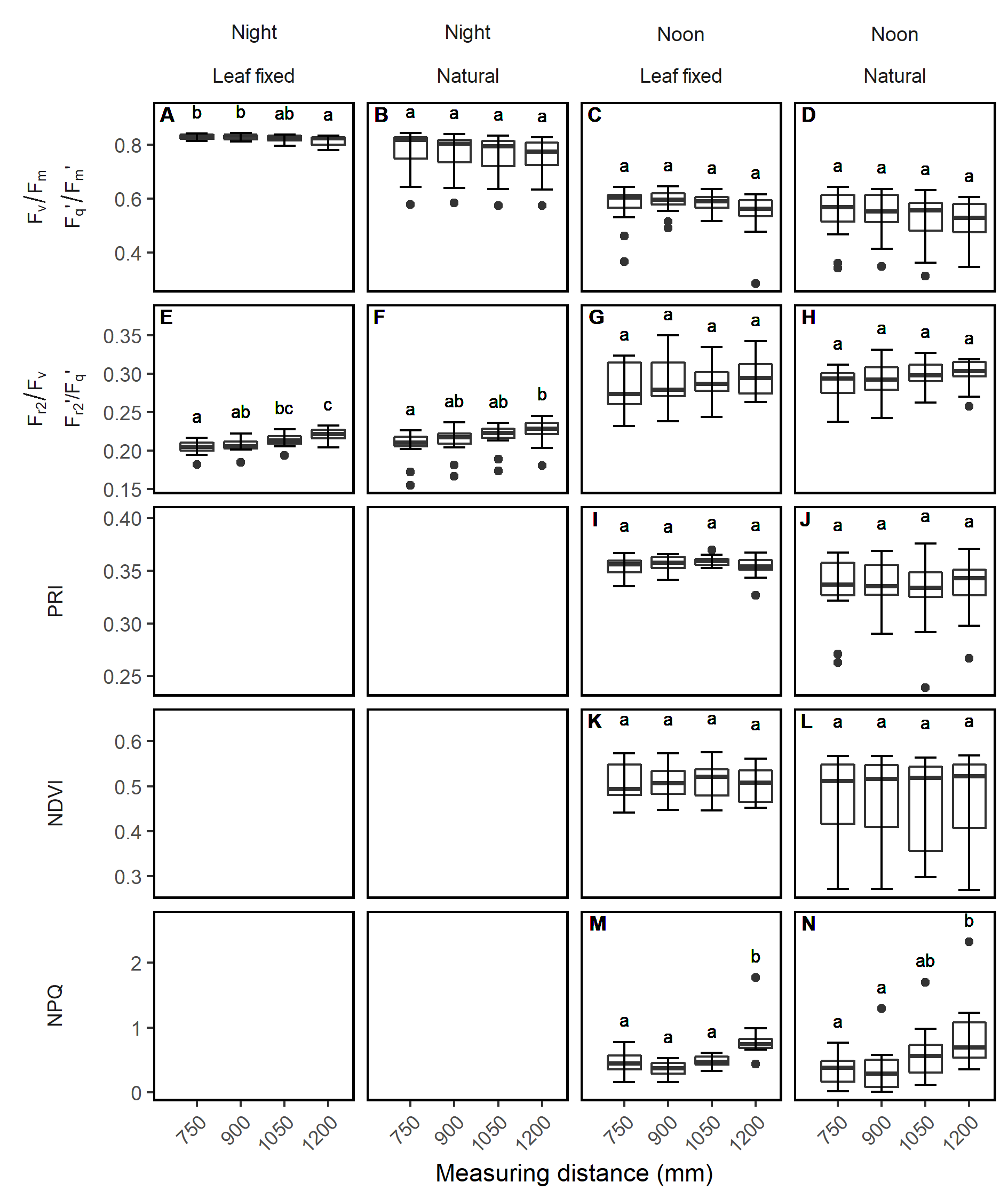


Supplementary Figure 7

Boxplot of operating efficiency of the photosystem II (F_v_/F_m_ in the dark and F_q_’/F_m_’ in the light, A to D) and reoxidation efficiency 5 ms after primary quinone (Q_A_) reduction (F_r2_/F_v_ in the dark and F_r2_’/F_q_’ in the light, E to H), photochemical reflectance index (PRI, I and J), normalized difference vegetation index (NDVI, K and L) and non-photochemical quenching (NPQ, M and N) in soybean genotype Tourmaline. Measuring distance ranged from 750 mm to 1200 mm and leave angles were fixed horizontal or in natural orientation. Measurements were carried out at night or at noon (n=14 to 16). Box represents inter-quartile range, bold horizontal bar the median, the discontinuous lines the upper and lower quartile, and outlier data points (>1.5 × inter-quartile range) are depicted by a point. Means with different letters differ significantly (p<0.05) using one way ANOVA followed by Tukey’s multiple comparisons of means.

# Supplementary Table

Supplementary Table 1

List of measured soybean genotypes, their source along with information available on cold tolerance and cultivar registration

| Genotype | Source | Cold tolerance | Registered in |
| --- | --- | --- | --- |
| Amarok | Swiss soybean breeding program of Agroscope | yes | Germany |
| Gallec |  | yes | Switzerland |
| Tourmaline |  | yes | Switzerland |
| 22216 |  | no | - |
| S1 |  | no | Canada |
| Protibus |  | no | Switzerland |
| MinnGold | University of Udine, Italy |  |  |
| Bahia |  |  |  |
| Eiko | Asgrow, USA |  |  |

Supplementary Table 2

List of measured maize genotypes, their source and the year when the genotypes were monitored

| Genotype | Source | 2016 | 2017 |
| --- | --- | --- | --- |
| PHT77  SO52  P148  B73  N22  PO74  W117  B106  P135  EC334 | IPK Gatersleben | no  yes  no  yes  yes  no  no  yes  no  yes | yes  yes  yes  yes  no  yes  yes  yes  yes  yes |
| Mo17 | University of Bonn | yes | no |

Supplementary Table 3

Liner model of photosystem II operating efficiency (F_q_’/F_m_’) measured in four crop species over two seasons in an unheated glasshouse (n= 283,730). Depending factors or covariates were photochemical reflectance index (PRI), photosynthetic photon flux density (PPFD), pseudo normalized difference vegetation index (pNDVI), alternative pNDVI (pNDVI_II), date and month of measurement, genotype, crop species, green normalized difference vegetation index (GNDVI), normalized difference vegetation index (NDVI), humidity and temperature. Descriptors are degree of freedom (Df), sum of squares (Sum Sq), mean of squares (Mean Sq), ratio of Mean Sq and Mean Sq error (F value) and the explained Sum Sq per factor (ExpVar). F_q_’/F_m_’ with associated PPFD < 25 were excluded.

|  | Df | Sum Sq | Mean Sq | F value | ExpVar  (%) |
| --- | --- | --- | --- | --- | --- |
| Residuals | 283540 | 1327.59 | 0 | NA | 39.5 |
| PRI | 1 | 707.01 | 707.01 | 150998.5 | 21.1 |
| PPFD | 1 | 404.46 | 404.46 | 86382.4 | 12 |
| pNDVI_II | 1 | 197.6 | 197.6 | 42201.8 | 5.9 |
| Date | 95 | 145.74 | 1.53 | 327.7 | 4.3 |
| pNDVI | 1 | 134.06 | 134.06 | 28632.9 | 4 |
| PPFD^0.5^ | 1 | 124.13 | 124.13 | 26511.3 | 3.7 |
| Plot | 46 | 68.47 | 1.49 | 317.9 | 2 |
| Month | 11 | 66.73 | 6.07 | 1295.6 | 2 |
| Genotype | 24 | 46.78 | 1.95 | 416.3 | 1.4 |
| Crop | 3 | 42.53 | 14.18 | 3027.6 | 1.3 |
| GNDVI | 1 | 37.16 | 37.16 | 7936.4 | 1.1 |
| NDVI | 1 | 26.11 | 26.11 | 5576.8 | 0.8 |
| Humidity | 1 | 18.51 | 18.51 | 3953 | 0.6 |
| Temperature | 1 | 11.27 | 11.27 | 2406.2 | 0.3 |

Supplementary Table 4

Coefficients of ridge regression parameters for the estimation of quantum efficiency of the photosystem II (F_v_/F_m_ in the dark and F_q_’/F_m_’ in the light) and the efficiency of electron transport 5 ms after reduction of primary quinone (Q_A_) (F_r2_/F_v_ in the dark, F_r2_’/F_q_’ in the light) for a full season where only environmental predictors are known.

See additional file “Data Sheet 2.CSV”

Supplementary Table 5

Analysis of variance (ANOVA) was carried out for signal to noise ratio (S/N ratio), maximum quantum efficiency of the photosystem II (F_v_/F_m_) and reoxidation efficiency 5 ms after primary quinone (Q_A_) reduction (F_r2_/F_v_) with distance from 750 mm to 1200 mm and fixed or natural leaf angle as depending factors. ANOVA is described by degree of freedom (Df), sum of squares (Sum Sq), mean of squares (Mean Sq), ratio of Mean Sq and Mean Sq error (F value), the associated p value (Pr(>F)) and the explained Sum Sq per factor (ExpVar). Measurements were done during the night (n=123).

|  | Variable | Df | Sum Sq | Mean Sq | F value | Pr(>F) | ExpVar |
| --- | --- | --- | --- | --- | --- | --- | --- |
| Distance | S/N ratio | 3 | 26464.13 | 8821.38 | 9.4 | 1.00E-05 | 14.9 |
| Leaf_angle |  | 1 | 39819.06 | 39819.06 | 42.3 | 0 | 22.4 |
| Residuals |  | 118 | 111170.81 | 942.13 | NA | NA | 62.6 |
| Distance | F_v_/F_m_ | 3 | 0.01 | 0 | 0.7 | 0.53691 | 1.4 |
| Leaf_angle |  | 1 | 0.11 | 0.11 | 37.2 | 0 | 23.6 |
| Residuals |  | 118 | 0.35 | 0 | NA | NA | 75 |
| Distance | F_r2_/F_v_ | 3 | 0.01 | 0 | 9.7 | 0.00001 | 19.3 |
| Leaf_angle |  | 1 | 0 | 0 | 3.4 | 0.06953 | 2.2 |
| Residuals |  | 118 | 0.02 | 0 | NA | NA | 78.4 |

Supplementary Table 6

Analysis of variance (ANOVA) was carried out for signal to noise ratio (S/N ratio), maximum efficiency of the photosystem II (F_q_/F_m_), reoxidation efficiency 5 ms after primary quinone (Q_A_) reduction (F_r2_/F_q_) and photochemical reflectance index (PRI) with distance from 750 mm to 1200 mm and fixed or natural leaf angle as depending factors. ANOVA is described by degree of freedom (Df), sum of squares (Sum Sq), mean of squares (Mean Sq), ratio of Mean Sq and Mean Sq error (F value), the associated p value (Pr(>F)) and the explained Sum Sq per factor (ExpVar). Measurements were done at noon (n=124).

|  | Variable | Df | Sum Sq | Mean Sq | F value | Pr(>F) | ExpVar |
| --- | --- | --- | --- | --- | --- | --- | --- |
| Distance | S/N ratio | 3 | 62982.91 | 20994.3 | 5 | 0.00252 | 7.9 |
| Leaf_angle |  | 1 | 236366.82 | 236366.82 | 56.8 | 0 | 29.7 |
| Residuals |  | 119 | 495334.64 | 4162.48 | NA | NA | 62.3 |
| Distance | F_q_’/F_m_’ | 3 | 0.02 | 0.01 | 1.3 | 0.26894 | 3 |
| Leaf_angle |  | 1 | 0.06 | 0.06 | 10.6 | 0.00145 | 8 |
| Residuals |  | 119 | 0.64 | 0.01 | NA | NA | 89.1 |
| Distance | F_r2_’/F_q_’ | 3 | 0 | 0 | 1.9 | 0.13955 | 4.5 |
| Leaf_angle |  | 1 | 0 | 0 | 0.8 | 0.37409 | 0.6 |
| Residuals |  | 119 | 0.07 | 0 | NA | NA | 94.9 |
| Distance | PRI | 3 | 0 | 0 | 0 | 0.98784 | 0.1 |
| Leaf_angle |  | 1 | 0.01 | 0.01 | 35.1 | 0 | 22.7 |
| Residuals |  | 119 | 0.05 | 0 | NA | NA | 77.2 |
